# Exploring Phytoremediation Potential: A Comprehensive Study of Flora Inventory and Soil Heavy Metal Contents in the Northeastern Mining Districts of Morocco

**DOI:** 10.3390/plants13131811

**Published:** 2024-06-30

**Authors:** Mohammed Oujdi, Yassine Chafik, Azzouz Boukroute, Sylvain Bourgerie, Marta Sena-Velez, Domenico Morabito, Mohamed Addi

**Affiliations:** 1 Laboratory for Agricultural Productions Improvement, Biotechnology and Environment (LAPABE), Faculty of Sciences, University Mohammed First, BP-717, Oujda 60000, Morocco; boukroute@hotmail.com; 2 University of Orleans, P2E-EA1207, INRAE USC1328, Rue de Chartres, Cedex 2, 45067 Orleans, Francedomenico.morabito@univ-orleans.fr (D.M.)

**Keywords:** mine site, phytoremediation, flora inventory, hyperaccumulation, heavy metals

## Abstract

Mining activities produce waste materials and effluents with very high metal concentrations that can negatively impact ecosystems and human health. Consequently, data on soil and plant metal levels are crucial for evaluating pollution severity and formulating soil reclamation strategies, such as phytoremediation. Our research focused on soils and vegetation of a highly contaminated site with potentially toxic metals (Pb, Zn, and Cu) in the Touissit mining districts of eastern Morocco. Vegetation inventory was carried out in three mine tailings of the Touissit mine fields using the “field tower” technique. Here, 91 species belonging to 23 families were inventoried: the most represented families were *Poaceae* and *Asteraceae*, and the biological spectrum indicated a predominance of Therophytes (55.12%). From the studied areas, 15 species were selected and collected in triplicate on the tailings and sampled with their corresponding rhizospheric soils, and analyzed for Pb, Zn, and Cu concentrations. *Reseda lutea*, *lotus marocanus*, and *lotus corniculatus* can be considered as hyperaccumulators of Pb, as these plants accumulated more than 1000 mg·kg^−1^ in their aerial parts. According to TF, these plant species could serve as effective plants for Pb phytoextraction.

## 1. Introduction

The degradation of terrestrial resources resulting from mining activities presents a serious threat to the environment. It has been estimated that approximately 0.4 × 10^6^ km^2^ of land worldwide are affected by mining activities [1]. Indeed, extractive activities have significant repercussions on the environment, especially due to the mechanization of operations. Apart from the negative aesthetic impact, abandoned mine sites have poor soils and limited vegetation, and they are generally abiotic, highly susceptible to erosion, and likely to pollute a large surrounding area [2]. Mining activities involving copper, zinc, lead, manganese, and iron, commonly utilized in various industrial applications, have been identified as the primary sources of soil pollution [3]. High concentrations of these heavy metals have consistently been found in soil samples collected from mining sites, underscoring the need for a comprehensive evaluation of their diverse environmental impacts [4]. The contamination of soil by heavy metals due to mining activities poses significant environmental risks, including soil and water pollution, disruption of ecosystems, and the potential release of hazardous materials into the environment [5].

For this reason, it is crucial to restore productivity to affected lands and prepare them for revegetation after mineral extraction. Growing environmental concerns have made post-mining reclamation of degraded land an essential aspect of the entire mining process [6]. Soil remediation methods include physical, chemical, and biological remediation [7,8]. Physical and chemical methods present drawbacks, such as high costs, permanent alteration of soil properties, and secondary pollution [9,10]. Among these restoration approaches, phytoremediation stands out for its cost-effectiveness, environmental sustainability, and economic advantages [11]. Phytoremediation is one of the emerging techniques extensively utilized for soil remediation, aiming to eliminate and/or stabilize pollutants in the environment [12]. Its application extends beyond soil stabilization or pollution mitigation and can also enhance aesthetics [13]. However, a crucial aspect of phytoremediation is the selection of appropriate indigenous plant species that are well suited to the edaphic conditions of the environment, with high tolerance and metal accumulation capacity [14]. Natural succession on mining residues is typically slow, with low species richness and limited colonization remaining unchanged for decades or even centuries. However, mining residues have the ability to collect a unique assemblage of plant species, different from the surrounding vegetation. This species can play a significant role in informing ecological restoration practices [15]. Phytoremediation involves the natural capabilities of plants to absorb, accumulate, and detoxify heavy metals present in soil. Certain plant species, known as hyperaccumulators, possess the unique ability to accumulate high concentrations of metals in their tissues. By strategically cultivating these plants in contaminated areas, they can effectively absorb and sequester metals, reducing soil contamination. Once the plants have absorbed the metals, they can be harvested and removed, thereby eliminating the contaminants from the soil [16]. A variety of plant species are employed in phytoremediation processes to remove heavy metal ions, including *Brassica napus*, *Brassica juncea*, *Festuca arundinacea*, *Pinus massoniana*, *Phragmites australis*, *Medicago sativa*, *Lolium perenne*, *Robinia pseudoacacia*, *Brassica juncea* (Indian mustard), *Chrysopogon zizanioides* (Vetiver grass), Fern, *Sedum alfredii*, *Chrysopogon zizanioides*, and *Helianthus annuus* [17,18]. Phytoremediation encompasses a range of techniques tailored to different pollutants, such as phytoextraction, phytostabilization, and phytovolatilization [19]. Key factors influencing the effectiveness of phytoextraction include the bioconcentration factor and the translocation factor, which reflect the accumulation and movement of metals within plants, respectively. Enhancements such as chelators and soil conditioners can boost the phytoextraction efficiency by enhancing metal accessibility and mitigating metal toxicity [20].

The Oriental region of Morocco is known for its rich geological structure and is historically associated with the production of lead. The region has been a significant contributor to the national production of lead, with the construction of a foundry in Oued El-Heimer in 1945, located 33 km northeast of Oujda. In 1980, Morocco’s lead production reached 170,000 tons, accounting for 3.5% of the global production. In 1998, the foundry treated 92,666 tons of lead concentrate, with 79% coming from the Touissit, Tighza, and other mines [21]. The abandoned mining sites of Touissit, under the management of the CMT group (Compagnie Minière de Touissit), have been a serious environmental problem since the ore was exhausted in the 1990s, and are represented by the washing waste that forms sand dykes with high concentrations of heavy metals, particularly Pb, resulting in contamination of the region. Some dykes are covered by sterile or non-sterile materials, with or without plantations. Some dykes have been reforested with pine and acacia without prior phytoremediation studies. However, several species present in these sites are known to be hyperaccumulators, such as *Hirschfeldia incana* and *Heydesarum spinosissmum* [22]. It is, therefore, necessary to rehabilitate the abandoned mining site on a phytoremediation basis to prevent and control the impacts of the problems mentioned. The aims of this study are to (i) create an inventory of native flora growing naturally on this site and their taxonomy, and (ii) assess heavy metals’ contents in the soils in some dominant plants growing on abandoned Pb/Zn mining sites.

## 2. Results and Discussion

### 2.1. Flora Diversity and Composition

**Figure 1:**
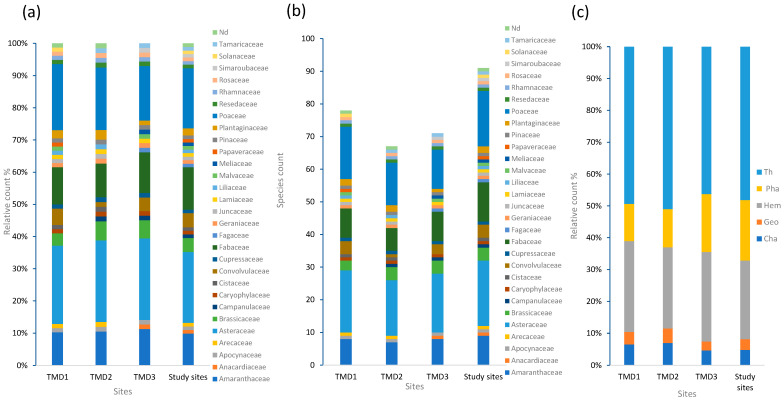
Contribution (**a**) and composition (**b**) of the families of species. (**c**) Biological spectrum of the vascular plants inventoried in different study sites.

Overall, 91 plants species were found in the Touissit mine tailings, belonging to 29 families (Figure 1), distributed over the 3 stations (TMD1, TMD2, and TMD3). The comparative study of the different surveys at the 3 mines tailings’ dykes showed a difference in composition and richness: floristic richness at the scale of each tailings’ dyke varied, with 78 (belonging to 23 families), 68 (belonging to 22 families), and 71 (belonging to 23 families), respectively, in TMD1, TMD2, and TMD3. Floristic richness remained remarkable compared with other mining sites, where richness and cover are very low. This is the case of the 5 tailing sites in southern China, with a richness of 56 species and a cover of just 3% [15], and the Lefke-Gemikonagi mine site in northern Cyprus, with just 23 species identified [14]. Additionally, 73 species (34 families) were found and mainly herbaceous plants in the wasteland of a pyrite mine in the southwest of Xingwen County, Sichuan Province, China [23]. This diversification of vegetation between tailings’ dykes could be due to the nature of the soil, which would experience varying degrees of contamination depending on the orientations as well as the age of the dykes [24]. The low level of natural colonization on mine waste dykes is mainly due to the difficulty of immigration of adapted species and the extreme physicochemical characteristics of these sites [14]. 

According to the analysis of the floristic composition (Figure 1a,b), *Asteraceae* and *Poaceae* families were the most prominently represented, showing a high percentage of 23.08% (21 species) and 18.68% (17 species), respectively, followed by *Fabaceae* at 13.04% (12 species), *Amaranthaceae* at 8.79% (8 species), *Convolvulaceae* at 4.40% (4 species), and *Brassicaceae* at 4.40% (4 species). Collectively, these families accounted for 66 species, representing 72.53% of the total recorded species. The remaining families exhibited a notably lower percentage and composition of species. The comparison of the results obtained for each studied station in terms of composition in botanical families and species (Figure 1a,b), taking into account the parameters of dyke reforestation and soil covering, highlighted that the stations richest in botanical families and species were TMD1 and TMD3, with 23 families, while TMD2 had only 22 families. The analysis of botanical families by station (Figure 1a,b) highlighted the existence of two classes of botanical families. First, typical families for each station, as follows: TMD1 station was characterized by *Papaveraceae* and *Solanaceae*, while TMD3 station differed by the presence of *Anacardiaceae*, *Fagaceae*, *Meliaceae*, and *Simaroubaceae*. Second, botanical families that were common to all stations included *Amaranthaceae*, *Apocynaceae*, *Asteraceae*, *Brassicaceae*, *Caryophylaceae*, *Geraniaceae*, *Convolvulaceae*, *Cupressaceae*, *Fabaceae*, *Lamiaceae*, *Pinaceae*, *Plantaginaceae*, *Poaceae*, *Resedaceae*, *Rhamnaceae*, and *Rosaceae.* The predominant botanical families in the weed flora were practically the same at all the mining dumps (TMD1, TMD2, and TMD3). Their manifestation and specific contribution to the weed flora slightly varied from one station to another. The dominance of these families is explained by their preponderance on the Morocco national scale, by their Mediterranean biogeographical range, and by their ability to adapt to unstable and diverse biotopes [25]. This is due to their high seed productivity and phenology, perfectly adapted to difficult edaphic conditions [26]. The dominance of the *Poaceae* and *Asteraceae* families suggests that these species have been able to adapt and tolerate unfavorable edaphic conditions (heavy metal toxicity, lack of nutrients, etc.). Metal tolerance has been demonstrated in numerous vascular plant families worldwide, and the majority of species widespread as metallophytes in the UK belong to three families: *Poaceae*, *Brassicaceae*, and *Caryophylaceae*. Other families, such as *Asteraceae*, *Rosaceae*, and *Apiaceae*, are poorly represented. Indeed, the successful colonization of *Poaceae* on current Pb/Zn mine tailings may reflect this strong potential for the evolution of metal tolerance [15].

Regarding the Ranaukier life-form of the 91 species (Figure 1c), the vegetation was dominated by *Therophytes* 55.12% (43 species), *Hemicryptophytes* 28.20% (22 species), *Phanerophytes* 21.79% (17 species), *Chamephytes* 5.49% (5 species), and *Geophytes* 3.84% (3 species). The general pattern of biological type on the sites was *Th* > *Hem* > *Pha* > *Cha* > *Geo.* The composition of the flora in the biological groups (Figure 1c) was relatively similar at all three sites. Figure 1c represents the proportion (%) of each biological type across the three sites (TMD1, TMD2, and TMD3). Similar to previous studies [27], the simple composition of the plants was probably caused by low soil fertility, which limited plant growth [28]. Zhu [29] reported that wasteland vegetation predominantly consists of herbaceous plants, comprising 94.1% of the flora. This observation highlights the survival characteristics of herbs, and with fine and light seeds quantity, easy propagation, and fast germination, herbaceous plants can easily survive in the contaminated areas [29,30]. In addition, herbaceous plants have developed roots, rapid growth rates, and the ability to grow in conditions of infertility, thus enhancing their ability to develop heavy metal tolerance [31].

### 2.2. Analysis of Freqeuncy and Coverage of Species

**Figure 2:**
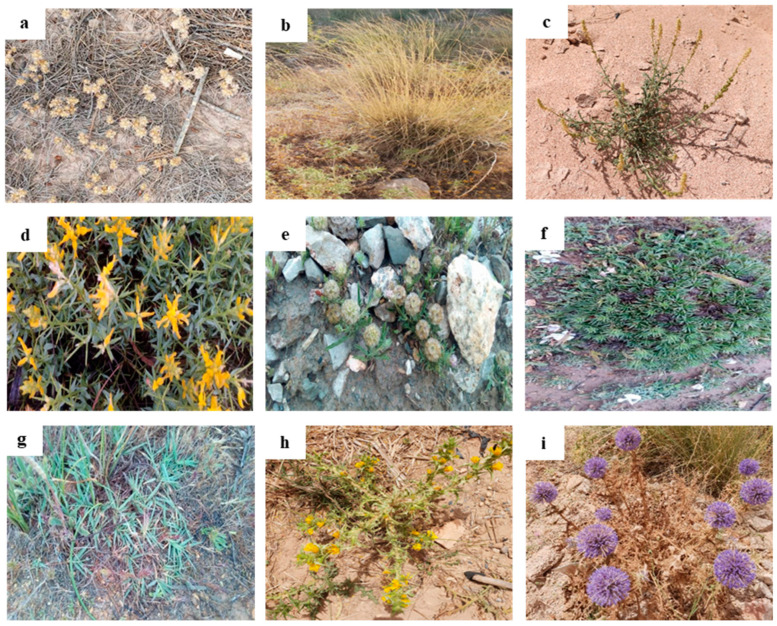
Illustrative images of some the herbaceous plants with the highest frequency Classes VI and III: (**a**) *Bromus hordeaceus* subsp. *mollis* (L.) Maire, (**b**) *Arisitda pungens Desf*., (**c**) *Reseda lutea* L., (**d**) *Genista hirsuta Vahl*, (**e**) *Lomelosia stellata* (L.) *Raf*., 1., (**f**) *Atractylis caepistosa Desf*., (**g**) *Cynodon dactylon* (L.) *Pers.*, (**h**) *Scolymus hispanicus* L., *1753.*, and (**i**) *Echinops spinosus* L.

Analysis of the relative frequency of species (Table 1) revealed four classes of species:

- (S) Class IV (Figure 2) (between 60% and 80%) contained 3 species: *Bromus mollis*, *Reseda lutea*, and *Aristida pungens*.
- Class III (Figure 2) (between 40% and 60%) contained 14 species, including *Atractylis caepistosa*, *Lamarckia aurea*, *Lomelosia stellata*, *Agatophora alopecoïdes*, *Carlina racemosa*, *Genista hirsuta*, *Phragmites australis*, *Astragatlus armatus*, and *Lotus maroccanus*.
- Class II (between 20% and 40%) comprised 31 species, including *Lotus corniculatus*, *Onopordum macracanthum schrub*, *Cardaria draba*, *Chenopodium murale*, *Genista trispucidata*, and *Juniperus oxycedrus*.
- Class I (−20%) comprised 49 plant species, including *Melia azedarach* L. *Pistacia lentiscus*, *Quercus ilex*, and *Retama monosperma*.

The most dominant plant species recovery indices, with D ≥ 200 (Table 1), were found for two plant species in the entire study area, with a maximum cover of D = 462 for *Aristida pungens*, followed by *Bromus mollis* with a cover value of D = 331. There were 10 species with D ≥ 100, namely, *Acacia cyanophylla*, *Genista hirsuta*, *Lotus corniculatus*, *Lotus maroccanus*, *Pinus halepensis*, *Arisitda pungens*, *Bromus mollis*, *Hordeum murinum*, *Lamarckia aurea*, and *Phragmites australis.*

The low level of natural colonization on mine waste dykes is mainly due to the difficulty of immigration of adapted species and the extreme physicochemical characteristics of these sites [14]. 

#### 2.2.1. Analyses of Species Composition in Different Sites

Analysis of vegetation diversity across various studied sites (Table 2 and Table S1) revealed distinct patterns in species abundance and ecosystem stability. 

At the TMD2 site, which contained the highest number of species (17 species with cover rate D ≥ 100), there was a substantial representation in ecological Classes V and III (28 species) and notable species richness (68 species), with the total coverage D = 5050 and sum of frequencies (%) = 2546.1, indicating a diverse and stable ecosystem due to successful revegetation efforts. TMD1, despite being older (24 years) and revegetated earlier with *Pinus* and *Acacia* species, displayed slightly lower diversity metrics (11 species > 100 in dominance, 11 species in Classes V and III, and species richness of 78). However, it maintains high diversity considering its smaller area (13.95 ha), with a high sum of coverage (D = 3854) and frequencies (% = 1823). In contrast, TMD3, the youngest site at 16 years, with revegetation starting in 2008 and the largest area (20.49 ha), showed fewer species exceeding 100 in dominance (7 species), lower representation in Classes V and III (10 species), and moderate species richness (71 species). The corresponding sum of coverage (D = 3503) and sum of frequencies (2076.86) were also lower.

The most frequent species in the study area included *Reseda lutea*, *Bromus mollis*, and *Aristida pungens*. *Reseda lutea* was the most frequent at TMD1, whereas *Aristida pungens* and *Bromus mollis* were the most frequent at TMD2, with 84.62% occurrence, followed by *Atractylis caepistosa*, *Astragalus armatus*, and *Lamarckia aurea* at 76.92%. At TMD3, *Bromus mollis* and *Reseda lutea* were the most frequent, with a 71.79% occurrence. TMD2 also had the highest number of abundant species (17 species), with *Aristida pungens* being the most abundant (D = 458). TMD1 followed with 11 abundant species, where *Bromus mollis* was the most abundant (D = 565). TMD3 had only 7 abundant species, with *Aristida pungens* remaining the most abundant (D = 835). Blońska et al. [32] found that mining activities can significantly diminish species richness. 

However, the implementation of revegetation strategies can enhance both the coverage per hectare and the species count. The higher species richness observed in TMD3 compared to TMD2, despite TMD2 being older and revegetated, may be due to the larger surface area of TMD3. Despite TMD3 having lower cover and frequency of vegetation, its greater area potentially supports more diverse habitats and ecological niches, contributing to increased species diversity.

These findings are consistent with Hendrychová’s study [33], which suggested that species composition within the community is positively influenced by revegetation efforts, contingent upon the age and species composition of the planted vegetation. Implementing vegetation in post-mining revegetation activities offers significant ecological benefits, including expediting ecological succession, providing habitat structure, and facilitating land rehabilitation to mitigate mining-induced environmental damage [34].

The vegetation analysis revealed that the grass *Aristida pungens* emerged as the dominant species in several age classes of the revegetation process. *Aristida pungens*, a type of grass, is well-adapted to various ecological niches. It demonstrates the ability to thrive in challenging environments with low soil fertility, such as post-mining sites. This species was abundant across all sites studied, indicating its robust potential for ecological restoration and adaptation to disturbed landscapes. This grass species demonstrates robust growth and adaptation capabilities in harsh environments with low fertility, such as post-mining land [35].

The post-mining reclamation efforts can significantly transform natural forest ecosystems. This transformation, as noted by Holl [36], is indicated by increasing species diversity and richness, along with the colonization of new species, reflecting successful ecological succession and habitat restoration on reclaimed land.

#### 2.2.2. Local Plant Diversity: A Promising Avenue for Sustainable Mining Reclamation (AHC)

##### Ground Coverage and Frequency Analysis

**Figure 3:**
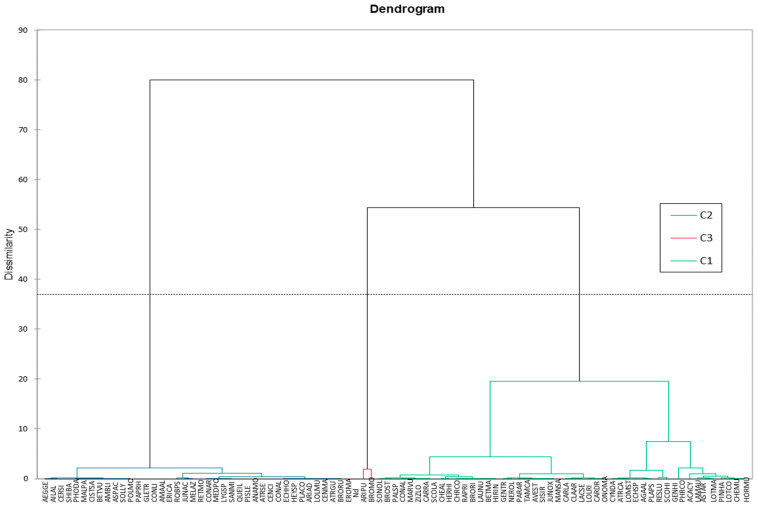
Cluster formation using the relative frequency and the cover values of plant species through the Ward D2 method [42]. C1: group 1, C2: group 2, and C3: group 3.

The relationship between average abundance and relative frequency provides an indication of the importance of a species [37]. Understanding the relationships between species’ frequency, abundance, and their clustering patterns is crucial for conservation efforts. Identifying key species that dominate or stabilize the ecosystem can inform management strategies aimed at preserving biodiversity and restoring disturbed environments, such as mining sites [38]. Thus, the relation between the species’ frequency and abundance was analyzed by PCA (Figure S1). Because of the large number of species, the species were grouped by cluster analysis into three groups (Figure 3). Each group consisted of species with very similar frequency and coverage parameters. To designate the principal species, priority was assigned to the frequency of a given species in its study area, while also considering its abundance [39].

The species of the group “a” were those with moderate frequency and abundance in all sites, and this group contained 48 species (Table S2). The broad range of distances within this group indicated a mix of species with high frequency and abundance. Species with large distances from the centroid (e.g., *Genista hirsute* and *Reseda lutea*) were likely to be dominant, suggesting they have adapted well to the mining environment and highlighting their competitive character and adaptability [40].

The group “b” was similar, but with lower frequency and abundance. This group was represented by 41 species. The low variance and small distances to the centroid implied that these species have more uniform frequency and cover rates, reflecting stable populations. These species might occupy more specific niches or be less competitive, indicating a more balanced presence within the site. Their balanced presence within the site indicated that they may be integral to maintaining ecological stability in these environments [38].

The group “c” had a notably higher frequency and abundance and was the most important in terms of species coverage and occurrence. Two species were included in this group, *Aristida pungens* and *Bromus mollis*. The significant distances from the centroid for these species indicated their high abundance and frequency, marking them as dominant species. Their dominant presence suggested that these species thrive particularly well under the current environmental conditions, making them ideal candidates for phytoremediation due to their robust growth and ability to establish quickly. *Aristida pungens* can be effectively used for stabilizing soil due to its robust root system that effectively anchors soil, preventing erosion and promoting stability [41].

### 2.3. Physicochemical Properties of Soil

The main physicochemical characteristics of mine tailings (presented in Table 3) revealed variations among the three sites (TMD1, TMD2, and TMD3). In our study, soils in all samples had neutral to alkaline pH, with values between 7.4 and 7.9. There was no significant difference (*p* < 0.0001). Similar to previous studies in the same mining area [22,43,44], this alkalinity generally results from the presence of carbonates in the soil. These carbonates can be an important reservoir for soil HMs. Pendias [45] reported that Cd, Cu, Pb, and Zn have a particularly high affinity for carbonate [22]. 

In terms of organic matter content (OM), the Touissit mine tailings generally had a very poor OM content, while TMD1, the oldest dyke with a decade of coverage and reforestation with pine, exhibited a slightly lower organic matter content (0.969%), but not statistically significant. In general, mine tailings have a low organic matter content [46]. 

The electrical conductivity (EC) of the tailings ranged from 129.2 to 293.9 µs·cm^−1^, being the highest in TMD3, probably due to the accumulation of salts at this site due to the absence of reforestation of the pond and the low cover rate by plant species. Several studies have shown that reforestation of mine tailings can lead to significant reductions in EC levels over time [47]. 

The total metal content in the different study sites was variable, ranging from 13,426.2 to 3417.8 mg·kg^−1^ for Pb, 7559.1 to 1906.4 mg·kg^−1^ for Zn, and 933.866 to 293.943 mg·kg^−1^ for Cu indicating the high heterogeneity of these mining wastes across the sites. The variation in ETM concentrations between sites, and even between different areas of the same site, seems to be a characteristic of pollution at mining sites [22]. Studies have consistently reported high concentrations of heavy metals at various mine sites. For example, El Hachimi et al. [48] reported significant contamination in the High Moulouya region of Morocco, with the Mibladen mine containing 10,520 mg Pb kg^−1^ and 9075 mg Zn kg^−1^, the Zaida mine containing 5547 mg Pb kg^−1^ and 7500 mg Zn kg^−1^, and the Aouli mine containing 2101 mg Pb kg^−1^ and 3125 mg Zn kg^−1^. 

The bio-extractible (Cacl2) Zn, Cu, and Pb showed low values regarding the values of pseudo-total concentrations. Furthermore, there were no differences between sites, expect for Pb, which showed slightly higher values ranging from 3.663 to 5.129 mg·kg^−1^. The highest values were in the TMD3 mine dump. Studies suggest that the bioavailability of lead (Pb) in neutral and alkaline sandy soils can be influenced by various factors, including soil properties, such as pH, organic matter content, and cation exchange capacity. Additionally, it should be noted that the availability of heavy metals, such as lead and zinc, is directly influenced by the minerals present in the soil, as well as other variables, such as pH [49]. The presence of specific minerals, such as anglesite, cerussite, and lead oxides, in Pb-contaminated soils and sediments can also reduce the bioavailability of lead [22,50]. Biochar amendment has been found to reduce the availability of Pb in the soil and its uptake in plants [47]. 

These findings highlight the complex interplay of various factors in determining the bioavailability of lead in neutral and alkaline sandy soils. The majority of abandoned mines are characterized by high levels of soil metal contamination due to anthropogenic activities. This has been supported by various studies, including research on the phytoremediation potential of native plant species in mine soils polluted by metal(loid)s and rare earth elements [51]. 

### 2.4. Heavy Metals in Rhizospheric Soils and Native Plants

Among the 15 studied plant species, there was variation in metal toxicity levels (Table 4) in the rhizospheric soil, with the highest concentrations observed in *Robinia pseudoacacia* for Pb with 29.61 g·kg^−1^, in *Quercus ilex* for Zn (20.02 g·kg^−1^), and in *Lotus maroccanus* for Cu (0.933 g·kg^−1^). In contrast, *Pistacia lentiscus* showed the lowest soil metal concentrations for Pb (2.39 g·kg^−1^), Zn (2.9 g·kg^−1^), and Cu (0.8 g·kg^−1^). *Astragalus armatus* showed the lowest Zn concentration in soils (1.74 g·kg^−1^). The metal kinetics in soil depends on factors such as intrinsic metal solubility, binding to soil particles, and soil physicochemical properties [52,53]. Root exudates influence metal dynamics in plants by altering the rhizospheric soil acidity, thereby enhancing pollutant availability to plants [54]. Specifically, variations in exudate effects among the studied plants may depend on root-system-specific development and the specific release of compounds, thereby modifying metal mobility in the soil [54].

Analysis of metal accumulation in plant species showed distinct patterns across Pb, Zn, and Cu accumulation in shoots and roots (Table 4). There were significant differences in heavy metals’ content between plant species (*p* < 0.0001). 

In shoots, the highest concentrations for all tested metals, Pb, Zn, and Cu, were in the aerial parts of *Lotus corniculatus*, *Lotus maroccanus*, and *Reseda lutea*, with 2270, 1480, and 1600 mg·kg^−1^ for Pb, 840, 1100, and 1060 mg·kg^−1^ for Zn, and 180.13, 221.32, and 167 mg·kg^−1^ for Cu, respectively, for the three plant species. These three species were the only plants studied that met the Pb hyperaccumulation criteria, which was 1000 mg·kg^−1^ for Pb and Cu and 10,000 mg·kg^−1^ for Zn in aerial parts, according to [55]. Matanzas et al. [51] proposed the use of *Lotus corniculatus* for phytostabilization of soil contaminated with heavy metals (Pb and As). Furthermore, endophytic bacteria associated with *Lotus corniculatus* have been studied for their hydrocarbon degradation potential and plant-growth-promoting activity, further highlighting the plants’ potential for phytoremediation [56]. *Reseda lutea* has been associated with phytoremediation processes, particularly in the context of zinc pollution in soil. Malayeri [57] have reported high zinc concentrations in *Reseda lutea* plants, indicating its potential for addressing zinc contamination in the environment. *Robinia pseudoacacia* exhibited the lowest foliar concentration of Pb (49.16 mg·kg^−1^), although it had the highest rhizhospheric lead concentration. Also had the lowest concentrations of Zn and Cu in shoots, with 72.01 mg·kg^−1^ of Zn and 7.85 mg·kg^−1^ of Cu. *Robinia pseudoacacia*, also known as black locust, has been identified as a potential metal excluder in some studies. This plant species exhibits an excluder phenotype for certain heavy metals, including cadmium (Cd), zinc (Zn), and lead (Pb). Additionally, *Robinia pseudoacacia* was found as a good extractor of arsenic (As) and boron (B) from the soil, indicating its ability to accumulate these elements in its leaves [58]. 

In roots, the lowest values were recorded for *Genista hirsuta* for all tested metals, with foliar metal concentrations of 152.99, 71.15, and 18.25 mg·kg^−1^ for Pb, Zn, and Cu, respectively. Anawar [59] reported that *Genista hirsuta* is not suitable for phytoremediation but may have major importance for the rehabilitation and recovery of the contaminated mining area as a metal excluder. In contrast, *Lotus maroccanus* accumulated the highest amount of Pb (0.86 g·kg^−1^) and Cu (139.08 mg·kg^−1^), while for Zn, *Aristida pungens* accumulated it in the roots more than the other species, with 1.30 g·kg^−1^.

However, it is essential to acknowledge that variations in metal accumulation patterns within the same plant species can be attributed to the distinct environmental conditions encompassing climate, soil characteristics, and the overall ecosystem. For instance, the climate in the Touissit region is characterized as semi-arid since it is heavily influenced by the Sahara [60], creating extreme conditions that may lead to varying behaviors within and across species in terms of biomass production and, consequently, metal accumulation. The recognition of indigenous plant species and the assessment of their capacity to tolerate and accumulate heavy metals are acknowledged as effective strategies for rehabilitating polluted environments [61]. Moreover, investigations into the native and prevalent flora thriving in abandoned Pb/Zn mining sites in eastern Morocco have enriched our knowledge of heavy metal tolerance and accumulation, underscoring the promise of phytoremediation [62,63].

### 2.5. Bioconcentration (BCF), Bioaccumulation Factor (BAC), and Translocation Factor (TF) of the Native Plants and Phytoremediation Potential

**Figure 4:**
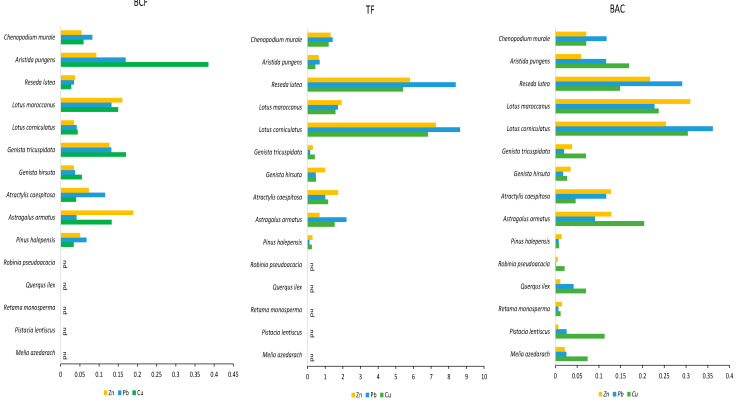
Bioconcentration factor (BCF), translocation factor (TF), and biological accumulation coefficient (BAC) for the studied plant species. Notes: red line indicates values TF > 1. nd mean not determined.

Results of TF, BCF, and BAC for Zn, Cu, and Pb are presented in Figure 4 and Table S3. In this study, TF, BCF, and BAC values exhibited variation among plant species and heavy metals. Translocation factors (TF) and bioconcentration factors (BCF) were employed to assess the efficacy of metal accumulation in plants and estimate their potential for phytoextraction and/or phytostabilization [64]. BCF, TF, and BAC were used to estimate the ability of plants to accumulate metals and to determine their phytostabilization and/or phytoextraction potential. The plants could be classified into four classes: high accumulator plants (1.0–10), moderate accumulator plants (0.1–1.0), low accumulator plants (0.01–0.1), and non-accumulator plants (<0.01) [65,66]. If BCF, TF, and BAC > 1, it means that the plant is a phytoextractor, while values of BCF > 1 and TF < 1 are criteria for assessing the phytostabilization potential of plants [44]. 

The majority of the examined plant species demonstrated TF > 1, indicating their capacity to translocate metals from roots to shoots, thus showcasing their potential for phytoextraction [67]. For the Touissit mine, five species, which were *Lotus corniculatus*, *Lotus maroccanus*, *Reseda lutea*, *Chenopodium murale*, and *Atractylis caespitosa*, showed a good ability of phytoremediation of three metals, Pb, Zn, and Cu, with a TF > 1. None of the plant species had a BCF or BAC higher than 1, which is in accordance with the findings of [44]. 

The highest values for TF were noted in *Lotus corniculatus* for Pb, Zn, and Cu (respectively, 8.62, 7.26, and 6.8), and *Reseda lutea* (8.38, 5.80, and 5.40). The species with TF > 1 for at least one metal were *Astragalus armatus* (Cu: 1.53 and Pb: 2.21), *Atractylis caespitosa* (Cu: 1.17, Pb: 1.01, and Zn: 1.74), *Genista hirsuta* (Cu: 1.81 and Zn: 1.39), *Lotus corniculatus* (Cu: 8.62, Pb: 6.82, and Zn: 7.27), *Lotus maroccanus* (Cu: 1.58, Pb: 1.72, and Zn: 2.82), *Reseda lutea* (Cu: 5.41, Pb: 8.38, and Zn: 9.34), and *Chenopodium murale* (Cu: 1.19, Pb: 1.43, and Zn: 1.31). *Astragalus armatus* showed potential for phytoextraction of copper with values above 1 and a BAC of Cu: 0.20. *Atractylis caespitosa* can extract lead and zinc effectively, with a BAC of Pb: 0.12 and Zn: 0.13. Both *Lotus corniculatus* (BAC for Cu: 0.63, Pb: 0.62, and Zn: 0.69) and *Lotus maroccanus* (BAC for Cu: 0.27, Pb: 0.23, and Zn: 0.23) demonstrated strong phytoextraction capabilities for Cu, Pb, and Zn, indicated by their high TF values. *Reseda lutea*, due to its high values of TF for multiple metals, is more suited for phytoextraction due to its low values of BCF (Cu: 0.23, Pb: 0.54, and Zn: 0.34). Conversely, *Genista hirsuta* (with BCF of Cu: 0.05 and Zn: 0.05) and *Chenopodium murale* (with BCF of Cu: 0.06, Pb: 0.08, and Zn: 0.05) are not suitable for phytoextraction because of their low values. It should be noted that differences in metals’ accumulation and translocation were not only observed between species but also within each individual species regarding Pb, Zn, and Cu. These variances may be associated with the solubility and complexity of metals in the rhizospheric soil, thereby affecting the plants’ ability to uptake and transport metals within their systems [68,69,70]. Furthermore, the capacity of plants to absorb or adsorb metals in their root systems is a crucial factor, as roots may sometimes limit the transportation of metals into other parts of the plant [71].

Based on the translocation factor (TF) values, we evaluated the phytoremediation potential of various plant species. Table S4 lists the species along with their corresponding phytoremediation strategies for lead (Pb), zinc (Zn), and copper (Cu).

#### 2.5.1. Phytoextraction

An ideal candidate for phytoextraction should demonstrate a translocation factor (TF) > 1, indicating efficient movement of heavy metals from roots to shoots. Additionally, it should possess a high capacity to accumulate multiple heavy metals in its above-ground biomass, facilitating effective harvest using conventional agricultural methods. Furthermore, the plant should exhibit robust resistance to infections and adverse growth conditions, commonly found in contaminated environments [72]. 

From the 15 plants studied, the most important plant species that demonstrated phytoextraction potential were *Lotus corniculatus*, *Lotus maroccanus*, *Reseda lutea*, *Atractyis caepitosa*, and *Chenopodium murale*, which demonstrated promising potential for phytoextraction of the tested heavy metals Cu, Pb, and Zn, as indicated by their translocation factor (TF) values exceeding 1. *Lotus corniculatus* and *Lotus maroccanus* exhibited significant TF values for these metals, along with high concentrations in their shoots, qualifying them as efficient phytoextractors. *Reseda lutea* also showed high TF values for Cu (TF = 5.41), Pb (TF = 8.38), and Zn (TF = 5.80). 

Additionally, these species accumulated more than 1000 mg·kg^−1^ of Pb in their aerial parts (*Lotus corniculatus* (2279.72 mg·kg^−1^), *Lotus maroccanus* (1484.51 mg·kg^−1^), and *Reseda lutea* (1609.82 mg·kg^−1^)), surpassing the hyperaccumulation threshold, which makes them good candidates for Pb phytoextraction. According to Nouri et al. [73], *Reseda lutea* accumulated 1774 mg·kg^−1^ of Pb in the vicinity of the Ahangaran lead–zinc mine in Hamedan, Iran, surpassing the threshold of 1000 mg·kg^−1^. Hasnaoui et al. [44] found significant Pb accumulation (±832.4 mg·kg^−1^) in the aerial parts of *Lotus corniculatus* at the Touissit mine site, aligning with our finding on the phytoextraction potential of this species. 

Also, these species were found dominant and frequent in our study sites. *Lotus corniculatus* exhibited high cover (D = 112.82). Similarly, *Lotus maroccanus* showed high cover (D = 110.26) and frequency Class III. *Reseda lutea* demonstrated moderate to high cover (D = 60.26) and was the most frequent species in our study area (Class IV). These results suggest their high adaptability to these sites. 

The *Lotus* genus species are known for their robust biomass production and deep rooting capabilities, which enhance their capacity to accumulate metals from contaminated soil. Their adaptability to diverse environmental conditions makes them suitable candidates for ecological restoration and phytoremediation efforts, particularly in soils affected by nutrient deficiencies, salinity, drought, or contaminants [74]. *L. corniculatus* is a cosmopolitan species noted for its ecological plasticity, thriving across diverse environmental gradients. It is prominently employed in ecological restoration efforts targeting soils afflicted by nutrient deficiency, salinity, drought, or contaminants, owing to its robust adaptive traits [75]. 

#### 2.5.2. Phytostabilization (Metal Excluders)

Plants selected for phytostabilization should demonstrate minimal metal bioaccumulation in above-ground tissues, restricted metal translocation from roots to shoots, dense canopy and root systems, rapid growth, and high tolerance to metal pollutants and adverse environmental conditions. It is advantageous for these species to be indigenous to the region, facilitating straightforward establishment and upkeep in contaminated areas. Examples of suitable plants include those that establish a dense vegetative cover while maintaining the lowest concentrations of metals in their above-ground biomass [72,75]. 

From our study, several species emerged as potential species that can be used in phytostabilization, with TF values < 1, high concentrations in roots and soil, and with low values in aerial parts (Table 4, Figure 4). Species such as *Genista tricuspidata* and *Pinus halepensis* showed a good phytostabilization potential, with TF < 1, higher concentrations of Pb, Zn, and Cu in the roots, and low values in aerial parts. These species, with their substantial biomass (*Genista tricuspidata* D = 80.77; *Pinus halepensis* D = 142.31), can effectively immobilize metals in the root zone, reducing metal mobility and bioavailability in the soil, thereby contributing to the stabilization of contaminated sites [64]. 

In our study, *Aristida pungens* emerged as the predominant species for phytostabilization of Pb, Zn, and Cu, characterized by a translocation factor (TF) < 1. This indicates limited metal translocation from roots to shoots, effectively reducing metal bioaccumulation in above-ground tissues. With the highest cover rate (D = 462.82), *Aristida pungens* is a grass species noted for its dense canopy and extensive root system, essential traits that significantly enhance soil stabilization and erosion control in contaminated environments [41].

### 2.6. Evaluation of Plants’ Heavy Metal Accumulation (PCA)

**Figure 5:**
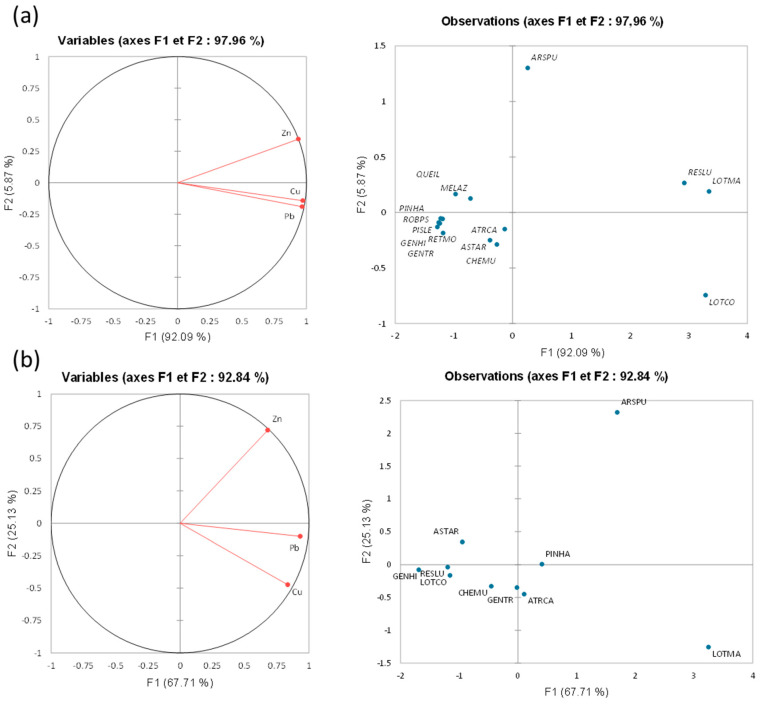
Plot of axes 1 and 2 of the principal component analysis (PCA), showing the means of the metal concentrations separately in the shoots (**a**) and the roots (**b**) for the studied species. Abbreviations of species are the same as in Table 1. Abbreviations are cited in Table 1.

The biplot derived from one-half of the principal component analysis (PCA), conducted on metal concentrations in the shoots of the studied plant species (Figure 5a), explained 97.96% of the total variance. The F1 axis, which accounts for 92.09% of the total inertia, signifies the species’ capability to simultaneously accumulate various heavy metals (Pb, Zn, and Cu). It distinguished *Reseda lutea*, *Lotus marrocanus*, and *Lotus corniculatus*, characterized by high concentrations of Cu, Zn, and Pb in their shoots, from the remaining 12 species, which exhibited low concentrations of these metals in their shoots (Figure 5a). These findings are consistent with prior research, suggesting that certain species have developed mechanisms to tolerate and accumulate elevated levels of heavy metals. This capability can be attributed to their distinctive physiological and biochemical properties [68,70]. The F2 axis, accounting for 5.87% of the total inertia, delineates the gradients of Pb, Zn, and Cu concentrations (Figure 5a). It distinguished *Arisitida pungens* and *Lotus corniculatus* from the other species. *Arisitida pungens* showed a strong correlation with Zn concentrations in shoots, whereas *Lotus corniculatus* exhibited a robust correlation with Pb and Cu. This indicates species-specific uptake and accumulation patterns, possibly attributable to variations in root exudates and metal transport mechanisms [76]. 

The biplot generated from one-half of the PCA conducted on metal concentrations in the roots of the studied species (Figure 5b) accounted for 92.84% of the total variance. The F1 axis, which represents 67.71% of the total inertia, predominantly explains the species’ capacity to simultaneously accumulate various heavy metals (Figure 5b). Along this axis, *Lotus maroccanus* and *Aristida pungens* exhibited a high correlation with Pb, Cu, and Zn, suggesting that these species possess strong accumulation abilities for all studied metals in their roots (Figure 5b). In contrast, the other group, consisting of *Pinus halepensis*, *Atractylis caepitosa*, *Genista tricuspidata*, and *Chenopodium murale* from another cluster, demonstrated a moderate accumulation of Pb, Zn, and Cu in their roots. The F2 axis (25.13%) for metal accumulation in roots adds another layer of understanding by distinguishing species based on their differential uptake of metals. This axis separates *Aristida pungens*, which was highly correlated with Zn accumulation, from *Lotus maroccanus*, which showed a strong correlation with Cu accumulation. These specific correlations suggest that *Aristida pungens* has a particular affinity for Zn, while *Lotus maroccanus* is more efficient at accumulating Cu. The species closer to the axis, such as *Pinus halepensis* and *Genista tricuspidata*, had lower concentrations of these metals in their roots, indicating weaker accumulation patterns. These differences can be attributed to variations in root morphology, exudate composition, and the presence of specific metal transporters and chelators that influence the bioavailability and uptake of metals [77]. The clusters of plant species identified with PCA were corroborated by a dendrogram obtained through ascending hierarchical classification (AHC; Figure S2).

These findings underscore the importance of species selection in phytoremediation strategies, where the goal is to enhance the removal of heavy metals from contaminated soils [64]. Future research should focus on further elucidating the mechanisms underlying metal uptake and accumulation in these species. Investigating the role of root exudates, mycorrhizal associations, and gene expression profiles related to metal transporters can provide deeper insights into the adaptive strategies of these plants [78]. Additionally, field trials should be conducted to assess the practicality and efficiency of these species in large-scale phytoremediation projects, considering factors such as soil type, metal speciation, and environmental conditions [79]. 

## 3. Materials and Methods

### 3.1. Description of the Study Site

**Figure 6:**
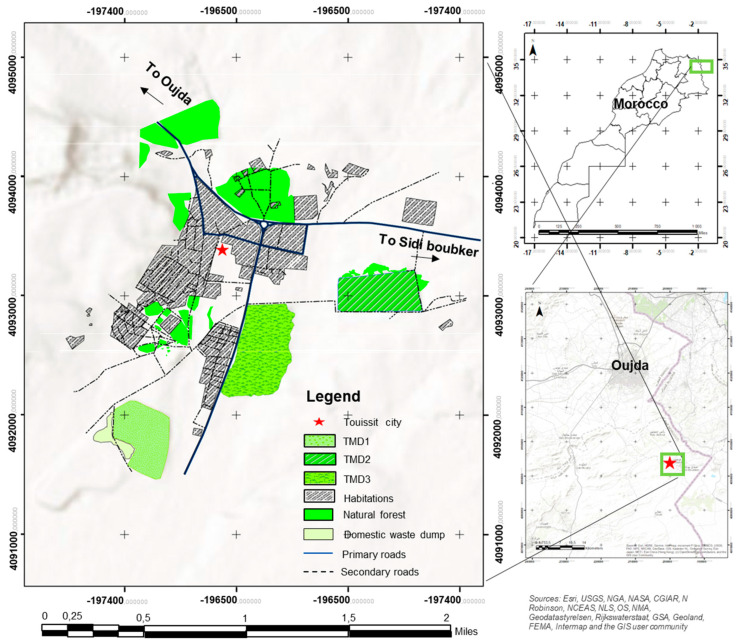
Location and description of the study sites in Touissit mining district.

The study site (Figure 6), located in the Touissit-Bou Beker district, lies in the northeastern portion of the horst range belt, covering an elongated, ENE-trending area spanning 64 square kilometers. It comprises five mines, with four situated in Morocco (Mekta, Beddiane, Touissit, and Bou Beker), and one in neighboring Algeria (El Abed). In this district, lead predominates in the western part, while zinc is more abundant in the eastern region. The Moroccan deposits, falling within a primary envelope of Pb + Zn mineralization established by a cutoff grade of 3% Pb + Zn, exhibit differences from most Mississippi Valley Type (MVT) deposits due to the prevalence of lead over zinc and the elevated concentrations of copper (Cu) at approximately 1% and silver (Ag) at 120 g/t. The Touissit Mining Company operated the Touissit mine from 1974 to 2002, resulting in the production of millions of tons of waste in the form of mill tailings and waste rock [80,81]. The Touissit-Boubker polymetallic district has produced 75 million tons of ores with 5% lead (Pb) and 3% zinc (Zn). This extensive mining operation has resulted in the generation of substantial quantities of both liquid and solid mining waste. The solid waste is currently stored in dykes situated near the city of Touissit. These dykes at the Touissit mine contain remnants of minerals leached by rainwater, raising potential concerns for human health and the nearby environment. Specifically, there are apprehensions regarding soil and water contamination [43,82,83].

The Touissit mining site is located 38 km south of the city of Oujda, and the mine is operated by the CMT Group (Mining Company of Touissit), a company that explores, extracts, and processes non-ferrous ores, namely, lead, zinc, and silver. Our study area is located in Eastern Morocco, which experiences a Mediterranean climate in the northern part of the region, transitioning to a much more arid and continental climate in the south, with a sunnier disposition. Average annual rainfall does not exceed 100 mm, which accounts for the overall scarcity of vegetation cover. The climate in the eastern region of Morocco is of the Mediterranean type, similar to the rest of Morocco. It is characterized by a dry summer of varying duration and a wet winter. Precipitation is low and distributed across three seasons (fall, winter, and spring). Overall, the amounts are not very substantial, and they often occur in the form of sudden showers [22,44,84]. The primary source of pollution in this region primarily comes from washing waste, forming substantial sand dykes. These sands can be readily carried by winds and precipitation in particulate form. It is essential to highlight the existence of numerous residences situated directly at the base of these dykes (Figure 6). Moreover, some of these dykes have been covered with a sterile substrate comprised of residual materials directly originating from the ore extraction activities conducted during the mine’s operation [22,82]. This study focused on three tailing deposits or mine dykes of Touissit mine districts, which differ in age and size (Figure 6). Work has been concentrated on these dykes because of their proximity to the town. The TMD1 dyke represents the oldest dyke in the Touissit region. Although covered for about a decade, it has undergone reforestation with pine and acacia. Dykes TMD3 and TMD2 represent an uncovered dyke and a covered dyke that have been reforested with pine and acacia, respectively (Table 5).

### 3.2. Sampling

#### 3.2.1. Flora Inventory

Among the various floristic sampling methods currently used, considering the nature of the mining dumps with sparse vegetation, we deemed it useful to employ the field tower survey sampling method [85]. This method considers the high heterogeneity and low density of vegetation distribution in the tailings, which allows for maximum comprehensiveness, enabling the identification of various species by surveying a significantly larger area than the minimum area specified by Braun-Blanquet [86]. This field survey method is the most comprehensive compared to other techniques for conducting vegetation surveys [87]. The method entails systematically traversing the area in various directions until no new species are encountered, which requires extensive coverage [88]. Additionally, as outlined in [85], this approach enables the inclusion of rare species, which hold significant agronomic importance, particularly those with rapid dissemination or those that serve as indicators of specific environmental characteristics [89].

**Figure 7:**
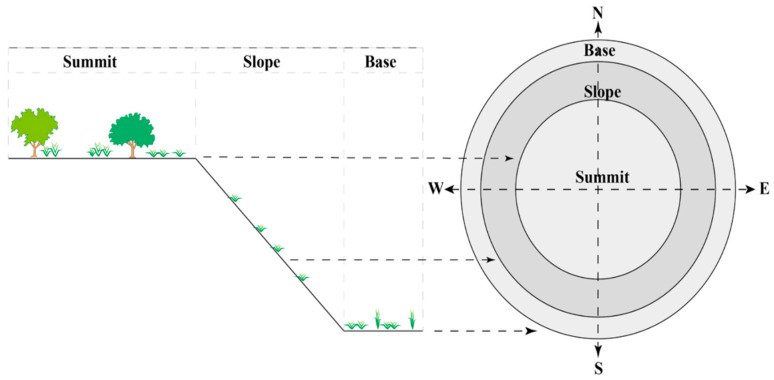
Basic scheme of flora inventory in the Touissit mine tailings.

The flora inventory of the tailings took place during the months of May, June, and July, in order to collect specimens during the flowering season, with a total of 39 plots (Figure 7): 13 plots per tailing across the 3 topographic horizons (base, slope, and summit). Each horizon was inventoried in accordance with the four cardinal directions (N, S, E, and W), with one transect for each cardinal direction and topographic horizon. Each transect was represented by one plot, and one plot was added at the summit center of each dump (Figure 7). For each recorded species, an abundance–dominance index was noted, which is represented through the 7-level Braun-Blanquet scale (r, +, 1, 2, 3, 4, and 5; here, this index was transformed to values for statistical treatment, to 0.5, 2.5, 15, 37.5, 62, 87.5, and 100%, respectively) [90]. The cover coefficient (D) was calculated by dividing the sum of the average percentage cover of species by the total number of phytosociological surveys and multiplying by 100 [91].

Relative frequency (RF) was calculated by dividing the number of hits where the species were recorded by the total number of plots. RF provides information on the rate of occurrence of a species along a transect for each site. The number and proportions of particular species were determined [92]. The proportion of each plant species was determined based on frequency classes (S): V—80–100% of all phytosociological relevés, IV—60–80%, III—40–60%, II—20–40%, and I—0.01–20% [91]. The inventoried plant species were also analyzed according to functional groups. Raunkiaer’s life-form system provides straightforward and effective indicators that characterize the botanical and ecological adaptations as well as habitat preferences of plants. The identified plant species were categorized into five primary life-form groups: Phanerophytes, Chamaephytes, Hemicryptophytes, Geophytes, and Therophytes. Assigning a life-form to each plant species facilitated the assessment of the proportion of different life-forms within the flora at the study sites, known as the biological spectrum. This spectrum is particularly valuable, as it reflects the vegetation structure and, consequently, offers insights into the climatic conditions of the surrounding environment [62]. From each site, three composite samples were randomly sampled for physicochemical analyses of tailings.

#### 3.2.2. Soil and Plant Sample Collection and Pretreatment

To assess the heavy metal content in both soil and plants, a total of 15 native dominant species (Table 6) were randomly collected, comprising 9 herbaceous species and 6 woody plants. These species were collected in triplicate from the tailings, along with their corresponding soil samples [93]. The concentrations of Pb, Zn, and Cu were analyzed for all samples, resulting in a total of 45 samples from dominant plants. To ensure accurate plant species information, soil samples were collected from the rooting zone (0–20 cm depth) of the plants. For woody species, only aerial parts (shoots) were sampled, along with their corresponding soil. All soil and plant samples were carefully sealed in clean polythene bags for transportation to the laboratory. In the laboratory, the fresh plants underwent meticulous washing with tap water, followed by three rinses with deionized water. Subsequently, the plant samples were oven-dried at 60 °C until a constant weight was achieved. The dried plant tissues were then separated into roots and shoots, milled into a fine powder, and stored in polythene bags for further analyses. The soil samples were air-dried, milled to a particle size less than 2 mm, and stored in polyethylene bags until subjected to analysis [94].

### 3.3. Chemical Analyses of Soil and Plant Samples

The pH and EC were determined with AFNOR standard NF T01-013. Here, 3 g of each soil sample was immersed in 21 mL of Milli-Q water at room temperature and subjected to agitation for 4 h at 200 rpm. Following filtration, pH and EC were determined using a combined pH–EC meter (Seven Excellence, Mettler-Toledo AG, Urtenen-Schönbühl, Switzerland), standard-calibrated with pH 4.0 and pH 7.0 and a KCl standard solution with an electrical conductivity of 1430 μs·cm^−1^. Soil organic matter (OM) was calculated using the mass loss percentage after burning, as described in [95]. Bio-extractable concentrations of heavy metals were determined using calcium chloride as a selective extractant at 0.01 M, with a ratio of 1:20 soil extractant, adapted from [96]. The mixtures were shacked for 2 h (50 rpm at room temperature), then centrifuged (10 min at 3000× *g*). Ultimately, the solution was filtered using Whatman filters, and then 5 mL was acidified with 83 µL of HNO_3_ for ICP–AOS measurement.

The plants (both shoots and roots) were separated and dried in an oven at 60 °C for 72 h [97]. Subsequently, the plant samples underwent acid digestion in a microwave system: 6 mL of 65% HNO_3_ and 3 mL of 35% HCl were combined with 0.2 g of plant sample. The mixtures were then heated using a pressurized vacuum microwave system (Multiwave 3000; Anton Paar GmbH, Ostfildern, Germany) with a heating rate of 15 min up to 180 °C, followed by a 15 min resting period at 180 °C and a 15 min cooldown period. After cooling to room temperature, the samples were diluted in 30 mL of ultrapure water (18 MΩ cm) and filtered through a 0.45 μm nitrocellulose filter [98]. Concentrations of Pb, Zn, and Cu were determined using ICP–AES (Inductively Coupled Plasma Atomic Emission Spectroscopy; ULTIMA 2, HORIBA, San Francisco, CA, USA). The same procedure was employed to assess the metal content in soils.

### 3.4. Calculation of Phytoremediation Indices

The efficiency of plants in accumulating heavy metals from the soil was determined by calculating the bioaccumulation factor (BAF). Additionally, the translocation factor (TF) was computed to assess the plants’ capacity to translocate heavy metals from roots to shoots. Phytoremediation indices were derived using the following formulas [28]:

BCF = [C]metal plant root/[C]metal soil(1)

TF = [C]metal plant shoot/[C]metal plant root(2)

BAC = [C]metal plant shoot/[C]metal soil(3)

BCF, BAC, and TF are commonly employed to assess the effectiveness of heavy metal accumulation in plants and to estimate their potential for phytoextraction and/or phytostabilization [29,64]

### 3.5. Identification of Plant Species

The botanical determination of most species was conducted in situ. Unidentified species were preserved in a herbarium and later identified using various botanical determination keys, such as the flora in [99], the practical flora of Morocco [25,100], and the herbarium specimens of ISTA Zraib. In the course of this work, an herbarium was created, comprising a collection of plants found on the mine site.

### 3.6. Statistics

To estimate significant differences (*p* < 0.0001) at confidence level of 95%, one-way analysis of variance (ANOVA) with Tukey’s test was performed as a parametric test for normal data to assess median comparisons between plant soil characteristics and between plant shoots, roots, and soil. The similarity between species for their cover and frequency was studied using principal component analysis (PCA) and ascending hierarchical classification (AHC). To investigate the relationships between metal concentrations in shoots and roots of the studied plant species, a principal component analysis (PCA) was conducted. For mapping purposes, spatial analysis was conducted using ArcGIS 10.3 Desktop (Esri, 2010), with Google Maps (Google LLC, 2023) serving as the base layer. Data integration, processing, and cartographic design adhered to standard procedures. Statistical calculations were performed using XLSTAT 2023 (XLSTAT Statistical Software for Excel).

## 4. Conclusions

The comprehensive investigation of the floristic diversity in the Touissit mine tailings revealed a high plant biodiversity, with 91 species belonging to 29 taxonomic families, despite the severe environmental conditions. The prevalence of *Asteraceae* and *Poaceae*, along with the dominant presence of species such as *Bromus mollis*, *Reseda lutea*, and *Aristida pungens*, underscored their physiological and ecological adaptability to environments characterized by high heavy metal (Pb, Zn, and Cu) concentrations, and low organic content. Species such as *Bromus mollis*, *Reseda lutea*, and *Aristida pungens* showed robust adaptation and potential for phytoremediation, with high frequencies or cover rates. Among the 15 studied species for heavy metal accumulation, *Lotus corniculatus*, *Lotus maroccanus*, *Reseda lutea*, and *Atractylis caepitosa* were highlighted as crucial for phytoremediation strategies. They effectively accumulated Pb, Zn, and Cu in their biomass, with Pb levels exceeding 1000 mg·kg^−1^, suitable for metal extraction from soils. Conversely, *Aristida pungens*, *Genista tricuspidata*, and *Pinus halepensis* had high metal concentrations in roots, offering phytostabilization perspectives. 

Research at these sites is now exploring biochar amendment effects on enhancing soil fertility and metal bioavailability and mycorrhizal associations for nutrient uptake to optimize metal tolerance and uptake mechanisms in hyperaccumulators using these species. Implementing these strategies through field trials will advance sustainable phytoremediation solutions for restoring mining-impacted soils and improving ecosystem health.

## Figures and Tables

**Table 1:** List of plant species present in the Touissit mine dumps with their frequency and cover rate (D) (Class IV (frequency between 60% and 80%), Class III (between 40% and 60%), Class II (between 20% and 40%), and Class I (−20%)).

Family	Life-Forms	Code	Species	Frequency Class	Frequency %	Cover (D)
Amaranthaceae	*Hem*	AGAAL	*Agatophora alopecoïdes* (*Delile*) *Bunge*	III	43.59	85.90
	*Th*	AMAAL	*Amaranthus albus* L.	I	2.56	1.28
	*Pha*	ATRSE	*Atriplex semibaccatan R*. *Br*.	I	10.26	5.13
	*Hem*	BETMA	*Beta macrocarpa Guss*.	II	30.77	29.49
	*Hem*	BETVU	*Beta vulgaris* L.	I	5.13	2.56
	*Th*	CHEAL	*Chenopodium album* L.	II	23.08	50.00
	*Th*	CHEMU	*Chenopodium murale* L.	II	33.33	98.72
Anacardiaceae	*Pha*	PISLE	*Pistacia lentiscus* L.	I	10.26	5.13
Apocynaceae	*Pha*	NEROL	*Nerium oleander* L.	II	28.21	78.21
Arecaceae	*Pha*	PHODA	*Phoenix dactylifera* L.	I	5.13	2.56
Asteraceae	*Th*	AMBLI	*Amberboa lipii* L.	I	5.13	2.56
	*Th*	ANAMO	*Anacyclus monanthos* L.	I	10.26	5.13
	*Hem*	ATRCA	*Atractylis caepistosa Desf*.	III	48.72	62.82
	*Th*	ATRGU	*Atractylis gummifera* L.	I	12.82	6.41
	*Hem*	CARLA	*Carlina racemosa* L.	III	43.59	46.15
	*Th*	CARRA	*Carthamus lanatus* L., *1753*	II	20.51	8.97
	*Th*	CENMA	*Centaurea marocana Balt*.	I	12.82	6.41
	*Th*	CHRCO	*Chrysanthemum coronarium* L.	II	30.77	15.38
	*Th*	ECHSP	*Echinops spinosus* L.	III	51.28	88.46
	*Th*	ECHHO	*Echium horridum Batt*.	I	15.38	7.69
	*Th*	ERICA	*Eryngium campestre* L., *1753*	I	2.56	1.28
	*Th*	LACSE	*Lactuca serriola* L.	II	38.46	32.05
	*Hem*	LAUNU	*Launaea nudicaulis Hook*.*f*.	II	28.21	26.92
	*Th*	LOMST	*Lomelosia stellata* (L.) *Raf*., *1838*	III	46.15	60.26
	*Hem*	MANSA	*Mantisalca salmantica* (L.) *Briq*. *& Cavill*.	II	33.33	66.67
	*Hem*	ONOMA	*Onopordum macracanthum schrub sb*,	II	35.90	43.59
	*Hem*	PALSP	*Pallenis spinosa* (L.) *Cass*., *1825*	II	23.08	11.54
	*Hem*	SCOHI	*Scolymus hispanicus* L., *1753*	III	58.97	66.67
	*Th*	SCOLA	*Scorzonera laciniata* L.	II	20.51	8.97
	*Th*	SONOL	*Sonchus oleraceus* L.	II	25.64	12.82
Brassicaceae	*Geo*	CARDR	*Cardaria draba* L.	II	33.33	41.03
	*Th*	HIRIN	*Hirschfieldia incana* (L.) *W*.*D*.*J*.*Koch*	II	28.21	39.74
	*Th*	RAPRI	*Rapistrum rugosum* (L.) *All*.	II	28.21	14.10
	*Th*	SISIR	*Sisymbrium irio* L.	II	33.33	58.97
Campanulaceae	*Th*	HEYSP	*Herniaria hirsuta* L.	II	15.38	7.69
Caryophylaceae	*Hem*	PARAR	*Paronychia argentea Lam*., *1779*	II	30.77	56.41
Cistaceae	*Pha*	CISTSA	*Cistus salviifolius* L.	I	5.13	2.56
Convolvulaceae	*Hem*	CONAL	*Convolvulus althaeoides* L., *1753*	I	15.38	6.41
	*Hem*	CONAL	*Convolvulus arvensis* L.	II	20.51	23.08
	*Hem*	CONAR	*Convolvus lineatus* L.	I	7.69	3.85
	*Hem*	CONLI	*Foeniculum vulgare subsp*. *Vulgare*	I	2.56	1.28
Cupressaceae	*Pha*	JUNOX	*Juniperus oxycedrus* L.	II	33.33	67.95
Fabaceae	*Pha*	ACACY	*Acacia cyanophylla Lindl*.	II	20.51	143.59
	*Cha*	ASTAR	*Astragalus armatus Willd*.	III	41.03	97.44
	*Pha*	CERSI	*Cercis siliquastrum* L., *1753*	I	5.13	15.38
	*Cha*	GENHI	*Genista hirsuta Vahl*.	III	43.59	185.90
	*Cha*	GENTR	*Genista tricuspidata Desf*.	II	33.33	80.77
	*Pha*	GLETR	*Gledistia trianthos* L.	I	2.56	1.28
	*Th*	HERHI	*Hedysarum spinosissimum* L.	II	20.51	50.00
	*Hem*	LOTCO	*Lotus corniculatus* L.	I	35.90	112.82
	*Hem*	LOTMA	*Lotus maroccanus ball*.	III	41.03	110.26
	*Th*	MEDPO	*Medicago polymorpha* L., *1753*	I	7.69	3.85
	*Pha*	RETMO	*Retama monosperma* (L.) *Boiss*., *1840*	I	7.69	3.85
	*Pha*	ROBPS	*Robinia pseudoacacia* L., *1753*	I	12.82	75.64
Fagaceae	*Pha*	QUEIL	*Quercus ilex* L., *1753*	I	10.26	5.13
Georaniaceae	*Th*	EROMA	*Erodium malacoïdes*, *Willd*.	I	12.82	5.13
Juncaceae	*Geo*	JUNAC	*Juncus acutus* L.	I	10.26	48.72
Lamiaceae	*Hem*	MARVU	*Marrubium vulgare* L.	I	17.95	21.79
Liliaceae	*Cha*	ASPAC	*Asparagus acutifolius* L.	I	5.13	2.56
Malvaceae	*Th*	MALPA	*Malva parviflora* L.	I	5.13	2.56
Meliaceae	*Pha*	MELAZ	*Melia azedarach* L.	I	10.26	30.77
Papaveraceae	*Th*	PAPRH	*Papaver rhoeas* L.	I	2.56	1.28
Pinaceae	*Pha*	PINHA	*Pinus halepensis Miller 1768*.	II	33.33	142.31
Plantaginaceae	*Th*	PLACO	*Plantago coronopus* L.	I	15.38	7.69
	*Th*	PLAPS	*Plantago psyllium Moench 1794*	III	43.59	71.79
Poaceae	*Th*	AEGGE	*Aegilops Geniculata Roth*	I	7.69	16.67
	*Hem*	ARIAD	*Arisitda adscensionis Desf*.	I	12.82	19.23
	*Hem*	ARIPU	***Arisitda pungens Desf*.**	**IV**	**66.67**	**462.82**
	*Th*	AVEST	*Avena sterilis* L.	I	35.90	56.41
	*Th*	BROMO	***Bromus hordeaceus subsp*. *mollis* (L.) *Maire***	**IV**	**71.79**	**330.77**
	*Th*	BRORI	*Bromus rigidus Roth*.	II	25.64	25.64
	*Th*	BRORU	*Bromus rubens* L.	I	12.82	6.41
	*Th*	BROST	*Bromus sterilis* (L.) *Nevsk*	II	23.08	10.26
	*Th*	CYNDA	*Cynodon dactylon* (L.) *Pers*.	III	51.28	51.28
	*Th*	HORMU	*Hordeum murinum* L.	II	30.77	110.26
	*Th*	LAMAU	*Lamarckia aurea* (L.) *Moench*	III	46.15	130.77
	*Th*	LOLMU	*Lolium multiflorum Lam*.	I	12.82	19.23
	*Th*	LOLRI	*Lolium rigidum Gaudin*	II	35.90	16.67
	*Cha*	LYGSP	*Lygeum spartum* L.	I	10.26	3.85
	*Geo*	PHRCO	*Phragmites australis* (*Cav*.) *Trin*. *ex Steud*.	III	43.59	193.59
	*Th*	POLMO	*Polypogon monspeliensis* (L.) *Desf*.	I	2.56	1.28
	*Th*	SHIBA	*Schismus barbatus* (L.) *Thell*.	I	5.13	2.56
Resedaceae	*Th*	RESLU	***Reseda lutea* L.**	**IV**	**71.79**	**60.26**
Rhamnaceae	*Pha*	ZIZLO	*Zizyphus lotus* (L.) *Desf*.	I	17.95	8.97
Rosaceae	*Hem*	SANMI	*Sanguisorba minor* L. *1753*	I	10.26	5.13
Simaroubaceae	*Pha*	AILAL	*Ailanthus altissima* (*Mill*.) *Swingle*, *1916*	I	5.13	15.38
Solanaceae	*Th*	SOLLY	*Solanum lycopersicum* L.	I	2.56	1.28
Tamaricaceae	*Pha*	TAMCA	*Tamarix canariensis Willd*.	II	28.21	52.56
Nd	*-*	Nd	*Sp n*.*d*	I	12.82	5.13

Notes. *Th:* Therophytes, *Pha:* Phanerophytes, *Hem*: Hemicryptophytes, *Cha*: Chamephytes, and *Geo*: Geophytes.

**Table 2:** Species composition of the studied sites.

	TMD1	TMD2	TMD3	Study Sites
Number of species (D > 100)	11	17	7	10
Number of species in Classes V and III	11	28	10	17
Species richness	78	68	71	91
Sum of coverage (D)	3854	5050	3503	4130
Sum of frequency %	1823	2546.1	2076.86	2148.7
Age	24	22	16	-

**Table 3:** Soil properties of Touissit mine tailings.

Site		TMD1	TMD2	TMD3
Age		24	22	16
SOM%	Mean	0.969 a	1.024 a	0.945 a
	SD	0.00	0.02	0.12
pH	Mean	7.613 a	7.490 a	7.900 a
	SD	0.11	0.14	0.02
EC (µs·cm^−3^)	Mean	129.267 b	148.800 b	254.200 a
	SD	28.05	15.36	8.43
Cu (mg·kg^−1^)	Mean	818.616 b	293.943 c	933.866 a
	SD	14.21	8.41	35.08
Pb (mg·kg^−1^)	Mean	13,426.182 a	3417.775 c	6527.435 b
	SD	126.03	50.72	344.37
Zn (mg·kg^−1^)	Mean	7559.096 a	1906.363 c	3582.990 b
	SD	21.02	9.21	151.11
Bio-extractible Cu (mg·kg^−1^)	Mean	1.422 ab	1.709 a	1.360 b
	SD	0.04	0.09	0.07
Bio-extractible Pb (mg·kg^−1^)	Mean	3.663 a	4.582 a	5.129 a
	SD	0.21	0.03	0.66
Bio-extractible Zn (mg·kg^−1^)	Mean	1.234 a	1.073 a	1.243 a
	SD	0.02	0.03	0.24

Note. The results are presented as means (*n* = 3), with values in the same column followed by the same letter indicating no statistically significant difference (*p* < 0.0001). SOM% means soil organic matter.

**Table 4:** Heavy metal concentrations (mg·kg^−1^ DW) in the roots/shoots and rhizospheric soils of plants collected from the Touissit mine tailings.

	Pb						Zn						Cu					
Species Code	Shoots	SD	Roots	SD	Soil	SD	Shoots	SD	Roots	SD	Soil	SD	Shoots	SD	Roots	SD	Soil	SD
ARSPU	416.870 c	98.47	607.629 a	79.18	3590.337 de	126.22	832.202 abc	208.41	1307.998 a	278.25	14,070.731 b	545.46	16.740 b	3.09	38.123 b	14.14	99.029 d	4.39
ASTAR	501.871 c	433.13	227.424 a	62.20	5501.175 de	1481.05	224.157 cd	188.31	329.593 b	65.83	1743.064 f	70.60	42.600 b	32.21	27.766 b	4.95	209.078 cd	7.46
ATRCA	572.682 bc	254.65	568.098 a	227.83	4908.724 de	327.58	306.905 bcd	133.10	176.512 b	68.06	2408.230 f	80.37	50.218 b	25.22	42.964 b	19.66	1077.393 a	432.11
CHEMU	544.825 bc	129.76	381.351 a	110.75	4637.003 de	98.06	236.134 bcd	45.79	179.779 b	41.33	3324.001 ef	8.36	49.949 b	5.05	41.891 b	5.04	704.562 abcd	9.99
GENHI	72.560 c	19.75	152.993 a	77.50	4072.320 de	179.95	72.016 d	18.41	71.157 b	32.61	2064.299 f	67.59	8.850 b	0.97	18.257 b	7.07	329.657 bcd	7.14
GENTR	69.012 c	2.76	451.040 a	210.59	3417.775 de	50.72	72.894 d	6.07	242.356 b	95.84	1906.363 f	9.21	20.600 b	3.33	50.065 ab	21.98	293.943 cd	8.41
LOTCO	2279.724 a	284.91	264.210 a	90.77	6307.600 cde	233.68	846.271 ab	90.56	116.482 b	34.36	3333.050 ef	82.34	180.113 a	24.32	26.423 b	5.11	592.092 abcd	27.46
LOTMA	1484.512 ab	342.93	864.085 a	294.78	6527.435 cd	344.37	1109.958 a	307.16	575.610 b	245.35	3582.990 ef	151.11	221.332 a	64.48	139.809 a	48.11	933.866 ab	35.08
MELAZ	248.873 c	11.69	nd	nd	9844.292 bc	345.08	267.758 bcd	6.47	nd	nd	12,488.420 b	919.13	21.493 b	0.21	nd	nd	290.655 cd	4.37
PINHA	76.555 c	8.44	676.562 a	64.35	10,038.321 bc	921.77	88.972 d	2.69	312.924 b	27.83	6173.859 cd	169.38	7.877 b	0.40	31.630 b	2.30	924.319 ab	60.33
PISLE	61.991 c	1.78	nd	nd	2396.654 e	270.83	89.550 d	3.38	nd	nd	14,065.316 b	1063.89	11.710 b	0.37	nd	nd	103.779 d	17.01
QUEIL	151.694 c	5.01	nd	nd	3633.154 de	206.04	227.859 cd	2.29	nd	nd	20,028.993 a	543.59	8.797 b	0.16	nd	nd	125.885 d	9.41
RESLU	1609.817 a	154.11	192.046 a	35.47	5529.843 de	186.33	1063.421 a	89.08	183.274 b	31.86	4889.819 de	392.27	167.005 a	16.46	30.879 b	7.60	1124.250 a	125.75
RETMO	93.629 c	3.44	nd	nd	13,426.182 b	126.03	112.726 d	3.19	nd	nd	7559.096 c	21.02	9.753 b	0.45	nd	nd	818.616 abc	14.21
ROBPS	49.165 c	2.91	nd	nd	29,661.245 a	2313.91	105.421 d	3.90	nd	nd	19,042.828 a	350.10	11.789 b	0.46	nd	nd	556.457 abcd	11.92

Note. The results are presented as means (*n* = 3), with values in the same column followed by the same letter indicating no statistically significant difference (*p* < 0.0001). nd mean not determined.

**Table 5:** Description of the study sites.

Site *	Year of Soil Amendment	Year of Revegetation	Age	Area (ha)
**TMD1**	2000	2001	24	13.95
**TMD2**	2002	2003	22	13.92
**TMD3**	2008	Not revegetated	16	20.49

* TMD1,TMD2, and TMD3: Touissit mine dumps 1, 2, and 3.

**Table 6:** List of sampled plant species for evaluation of their phytoremediation potential.

Sampling Site	No.	Family Name	Scientific Name	Life-Form	Abbreviation	Replicates
**Touissit mine dumps**	1	Fabaceae	*Astragalus armatus*	Hc	ASTAR	3
	2	Asteracea	*Atractylis caespitosa*	Hc	ATRCA	3
	3	Fabaceae	*Genista hirsuta*	Ph	GENHI	3
	4	Fabaceae	*Genista tricuspidata*	Ph	GENTR	3
	5	Fabaceae	*Lotus corniculatus*	Ph	LOTCO	3
	6	Fabaceae	*Lotus maroccanus*	Th	LOTMA	3
	7	Resedaceae	*Reseda lutea*	Th	RESLU	3
	8	Poaceae	*Aristida pungens*	Hc	ARSPU	3
	9	Chenopodiaceae	*Chenopodium murale*	Th	CHEMU	3
	10	Meliaceae	*Mélia azedarach*	Ph	MELAZ	3
	11	Anacardiaceae	*Pistacia lentiscus*	Ph	PISLE	3
	12	Fabaceae	*Retama monosperma*	Ph	RETMO	3
	13	Fagaceae	*Quercus ilex*	Ph	QUEIL	3
	14	Fabaceae	*Robinia pseudoacacia*	Ph	ROBPS	3
	15	Pinaceae	*Pinus halpensis*	Ph	PINHA	3

## Data Availability

The raw data supporting the conclusions of this article will be made available by the authors upon request.

## Supplementary Materials

The following supporting information can be downloaded at: https://www.mdpi.com/article/10.3390/plants13131811/s1, Table S1: Species composition in different study sites; Figure S1: Principal component analysis (PCA) of the vegetation dataset highlighting the relative frequency and the cover values of plant species. Group (a) Colored in blue species with the lowest values., Group (b) Colored in green species with average values, Group (c) Colored in red species of with the highest values; Table S2. The species groups according Ward method cluster analysis for species (abbreviations of species names are listed in Table 3). Table S3. Bioconcentration Factor (BCF), Translocation Factor (TF), and Biological Accumulation Coefficient (BAC) of the Studied Plant Species.; Figure S2. Dendrogram Derived from Ascending Hierarchical Classification (AHC) of Metal Concentrations in Shoots (a) and Roots (b) of Investigated Plant Species: Species Name Abbreviations are listed in Table 2. C1: group 1, C2: group 2, C3: group 3, C4: group 4, C5: group5; Table S4. Plants phytoremediation strategy.
