# Supplementary material for: Exploring Phytoremediation Potential: A Comprehensive Study of Flora Inventory and Soil Heavy Metal Contents in the Northeastern Mining Districts of Morocco
Source: Plants (Basel). 2024 Jun 30;13(13):1811. doi: 10.3390/plants13131811 (PMC11244480; doi:10.3390/plants13131811)
Supplement: Supplementary file 1 [file plants-13-01811-s001.zip › plants-3062035-supplementary.pdf]

Table S1. Species composition in different study sites

				TMD1		TMD2		TMD3		Study sites		
**Familly**	**Life forms**	***Species***	**Code**	**(D)**	**S**	**(D)**	**S**	**(D)**	**S**	**(D)**	**S**	**Frequency class**
**Amaranthaceae**	Hc	*Agatophora alopecoïdes*	AGAAL	62	46,15	177	46,15	19	38,46	86	43,59	III
	Th	*Amaranthus albus L.*	AMAAL	-	-	4	7,69	-	-	1	2,56	I
	Ph	*Atriplex semibaccatan R. Br.*	ATRSE	12	23,08	-	-	4	7,69	5	10,26	I
	Hc	*Beta macrocarpa Guss.*	BETMA	54	23,08	19	38,46	15	30,77	29	30,77	II
	Hc	*Beta vulgaris L.*	BETVU	4	7,69	-	-	4	7,69	3	5,13	I
	Th	*Chenopodium album L.*	CHEAL	42	7,69	4	7,69	104	53,85	50	23,08	II
	Th	*Chenopodium murale L.*	CHEMU	135	7,69	100	46,15	62	46,15	99	33,33	II
**Anacardiaceae**	Pha	*Pistacia lentiscus*	PISLE	-	-	-	-	15	30,77	5	10,26	I
**Apocynaceae**	Ph	*Nerium oleander L.*	NEROL	50	23,08	138	46,15	46	15,38	78	28,21	II
**Arecaceae**	Ph	*Phoenix dactylifera L.*	PHODA	4	7,69	4	7,69	-	-	3	5,13	I
**Asteraceae**	Th	*Amberboa lipii L.*	AMBLI	-	-	8	15,38	-	-	3	5,13	I
	Th	*Anacyclus monanthos L.*	ANAMO	4	7,69	8	15,38	4	7,69	5	10,26	I
	Hc	*Atractylis caepistosa Desf.*	ATRCA	12	23,08	154	76,92	23	46,15	63	48,72	III
	Th	*Atractylis gummifera*	ATRGU	15	30,77	-	-	4	7,69	6	12,82	I
	He	*Carlina racemosa L.*	CARLA	4	15,38	35	69,23	100	46,15	46	43,59	III
	Th	*Carthamus lanatus*	CARRA	4	15,38	15	30,77	8	15,38	9	20,51	II
	Th	*Centaurea marocana Balt.*	CENMA	4	7,69	12	23,08	4	7,69	6	12,82	I
	Th	*Chrysanthemum coronarium L.*	CHRCO	19	38,46	23	46,15	4	7,69	15	30,77	II
	Th	*Cladanthus arabicus (L.) Cass., 1817*	CLAAR	4	7,69	23	46,15	73	69,23	33	41,03	III
	Th	*Echinops spinosissimus turra*	ECHSP	50	30,77	69	61,54	146	61,54	88	51,28	III
	Th	*Echium horridum Batt.*	ECHHO	8	15,38	-	-	15	30,77	8	15,38	I

	Th	*Eryngium campestre L., 1753*	ERICA	4	7,69	-	-	-	-	1	2,56	I
	Th	*Lactuca serriola L.*	LACSE	54	30,77	31	61,54	12	23,08	32	38,46	II
	Hc	*Launaea nudicaulis Hook.f.*	LAUNU	8	15,38	58	38,46	15	30,77	27	28,21	II
	Th	*Lomelosia stellata (L.) Raf., 1838*	LOMST	12	30,77	112	69,23	58	38,46	60	46,15	III
	Hc	*Mantisalca salmantica (L.) Briq. & Cavill.*	MANSA	4	15,38	92	30,77	104	53,85	67	33,33	II
	Hc	*Onopordum macracanthum schrub sb,*	ONOMA	8	15,38	54	30,77	69	61,54	44	35,9	II
	Hc	*Pallenis spinosa (L.) Cass., 1825*	PALSP	12	23,08	15	30,77	8	15,38	12	23,08	II
	Hc	*Scolymus hispanicus L., 1753*	SCOHI	23	53,85	23	46,15	154	76,92	67	58,97	III
	Th	*Scorzonera laciniata L.*	SCOLA	8	23,08	12	23,08	8	15,38	9	20,51	II
	Th	*Sonchus oleraceus L.*	SONOL	12	23,08	8	15,38	19	38,46	13	25,64	II
**Brassicaceae**	Gé	*Cardaria draba L.*	CARDR	100	53,85	12	23,08	12	23,08	41	33,33	II
	Th	*Hirschfieldia incana (L.) W.D.J.Koch*	HIRIN	-	-	104	53,85	15	30,77	40	28,21	II
	Th	*Rapistrum rigosum*	RAPRI	19	38,46	15	30,77	8	15,38	14	28,21	II
	Th	*Sisymbrium irio L.*	SISIR	154	53,85	8	15,38	15	30,77	59	33,33	II
**Campanulaceae**	Th	*Herniaria hirsuta L.*	HERHI	-	-	19	38,46	4	7,69	8	15,38	I
**Caryophylaceae**	Hc	*Paronychia argentea Lam.*	PARAR	104	38,46	58	38,46	8	15,38	56	30,77	II
**Cistaceae**	Ph	*Cistus salviifolius L.*	CISTSA	4	7,69	4	7,69	-	-	3	5,13	I
**Convolvulaceae**	Hc	*Convolvulus althaeoides L., 1753*	CONAL	12	30,77	-	-	8	15,38	6	15,38	I
	Hc	*Convolvulus arvensis L.*	CONAL	8	15,38	46	15,38	15	30,77	23	20,51	II
	Hc	*Convolvus lineatus L.*	CONAR	8	15,38	-	-	4	7,69	4	7,69	I

	Hc	*Foeniculum vulgare subsp. Vulgare*	CONLI	4	7,69	-	-	-	-	1	2,56	I
**Cupressaceae**	Ph	*Juniperus oxycedrus L.*	JUNOX	92	30,77	58	38,46	54	30,77	68	33,33	II
**Fabaceae**	Ph	*Acacia cyanophylla Lindl*	ACACY	292	15,38	138	46,15	-	-	144	20,51	II
	Ch	*Astragatlus armatus*	ASTAR	85	15,38	154	76,92	54	30,77	97	41,03	III
	Ph	*Cercis silliquastrum*	CERSI	-	-	-	-	46	15,38	15	5,13	I
	Ch	*Genista hirsuta*	GENHI	231	46,15	238	61,54	88	23,08	186	43,59	III
	Ch	*Genista tris*	GENTR	50	23,08	142	53,85	50	23,08	81	33,33	II
	Ph	*Gledistia trianthos. L*	GLETR	-	-	-	-	4	7,69	1	2,56	I
	Th	*Hedysarum spinosissimum L.*	HEYSP	135	30,77	8	15,38	8	15,38	50	20,51	II
	Hc	*Lotus corniculatus*	LOTCO	262	30,77	65	53,85	12	23,08	113	35,9	II
	Hc	*Lotus maroccanus ball,*	LOTMA	173	38,46	150	69,23	8	15,38	110	41,03	III
	Th	*Medicago polymorpha L., 1753*	MEDPO	12	23,08	-	-	-	-	4	7,69	I
	Ph	*Retama monosperma*	RETMO	12	23,08	-	-	-	-	4	7,69	I
	Ph	*Robinia pseudoacacia L., 1753*	ROBPS	-	-	-	-	227	38,46	76	12,82	I
**Fagaceae**	Ph	*Querqus ilex*	QUEIL	-	-	-	-	15	30,77	5	10,26	I
**Geraniaceae**	Th	*Erodium malacoïdes, Willd.*	EROMA	4	15,38	4	7,69	8	15,38	5	12,82	I
**Juncaceae**	Gé	*Juncus acutus L.*	JUNAC	8	15,38	138	15,38	-	-	49	10,26	I
**Lamiaceae**	Hc	*Marrubium vulgare L.*	MARVU	4	7,69	12	23,08	50	23,08	22	17,95	I
**Liliaceae**	Ch	*Asparagus acutifolius L.*	ASPAC	4	7,69	4	7,69	-	-	3	5,13	I
**Malvaceae**	Th	*Malva parviflora L.*	MALPA	4	7,69	-	-	4	7,69	3	5,13	I
**Meliaceae**	Ph	*Melia azedarach L.*	MELAZ	-	-	-	-	92	30,77	31	10,26	I
**Papaveraceae**	Th	*Papaver rhoeas L.*	PAPRH	4	7,69	-	-	-	-	1	2,56	I
**Pinaceae**	Ph	*Pinus halepensis Miller 1768.*	PINHA	4	7,69	408	61,54	15	30,77	142	33,33	II

**Plantaginaceae**	Th	*Plantago coronopus L.*	PLACO	4	7,69	19	38,46	-	-	8	15,38	II
	Th	*Plantago psyllium Moench 1794*	PLAPS	92	38,46	104	53,85	19	38,46	72	43,59	III
**Poaceae**	Th	*Aegilops geniculata Roth*	AEGGE	4	7,69	46	15,38	-	-	17	7,69	I
	Hc	*Arisitda adscensionis Desf.*	ARIAD	4	7,69	50	23,08	4	7,69	19	12,82	I
	Hc	*Arisitda pungens Desf.*	ARIPU	96	30,77	458	84,62	835	84,62	463	66,67	IV
	Th	*Avena sterilis L.*	AVEST	12	23,08	69	61,54	88	23,08	56	35,9	II
	Th	*Bromus hordeaceus subsp. mollis (L.) Maire*	BROMO	565	53,85	273	84,62	154	76,92	331	71,79	IV
	Th	*Bromus rigidus Roth.*	BRORI	8	15,38	65	53,85	4	7,69	26	25,64	II
	Th	*Bromus rubens L.*	BRORU	-	-	8	15,38	12	23,08	6	12,82	I
	Th	*Bromus sterilis (L.) Nevsk*	BROST	12	30,77	12	23,08	8	15,38	10	23,08	II
	Hc	*Cenchrus ciliaris L.*	CENCI	19	46,15	-	-	-	-	6	15,38	II
	Th	*Cynodon dactylon (L.) Pers.*	CYNDA	27	53,85	58	38,46	69	61,54	51	51,28	III
	Th	*Hordeum murinum L.*	HORMU	296	23,08	12	23,08	23	46,15	110	30,77	II
	Th	*Lamarckia aurea (L.) Moench*	LAMAU	4	7,69	323	76,92	65	53,85	131	46,15	III
	Th	*Lolium multiflorum Lam.*	LOLMU	42	7,69	12	23,08	4	7,69	19	12,82	I
	Th	*Lolium rigidum Gaudin*	LOLRI	23	53,85	15	30,77	12	23,08	17	35,9	II
	Ch	*Lygeum spartum L.*	LYGSP	12	23,08	-	7,69	-	-	4	10,26	I
	Gé	*Phragmites australis (Cav.) Trin. ex Steud.*	PHRCO	142	53,85	392	61,54	46	15,38	194	43,59	III
	Th	*Polypogon monspeliensis (L.) Desf.*	POLMO	4	7,69	-	-	-	-	1	2,56	I
	Th	*Schismus barbatus (L.) Thell.*	SHIBA	8	15,38	-	-	-	-	3	5,13	I
**Resedaceae**	Th	*Reseda lutea L.*	RESLU	35	76,92	65	53,85	81	84,62	60	71,79	IV

**Rhamnaceae**	Ph	*Zizyphus lotus (L.) Desf.*	ZIZLO	8	15,38	8	15,38	12	23,08	9	17,95	I
**Rosaceae**	Hc	*Sanguisorba minorL. 1753*	SANMI	4	7,69	8	15,38	4	7,69	5	10,26	I
**Simaroubaceae**	Ph	*Aillanthus altissima*	AILAL	-	-	-	-	46	15,38	15	5,13	I
**Solanaceae**	Th	*Solanum lycopersicum L.*	SOLLY	4	7,69	-	-	-	-	1	2,56	I
**Tamaricaceae**	Ph	*Tamarix canariensis Willd.*	TAMCA	-	-	62	46,15	96	38,46	53	28,21	II
**Nd**		*Sp n.d*	Nd	8	23,08	8	15,38	-	-	5	12,82	I

Notes: *Th: therophytes, Pha: Phanerophytes, Hem: hemicryptophytes, Cha: Chamephytes, Geo: geophytes.* (S): Freqeuncy class, (D): Cover

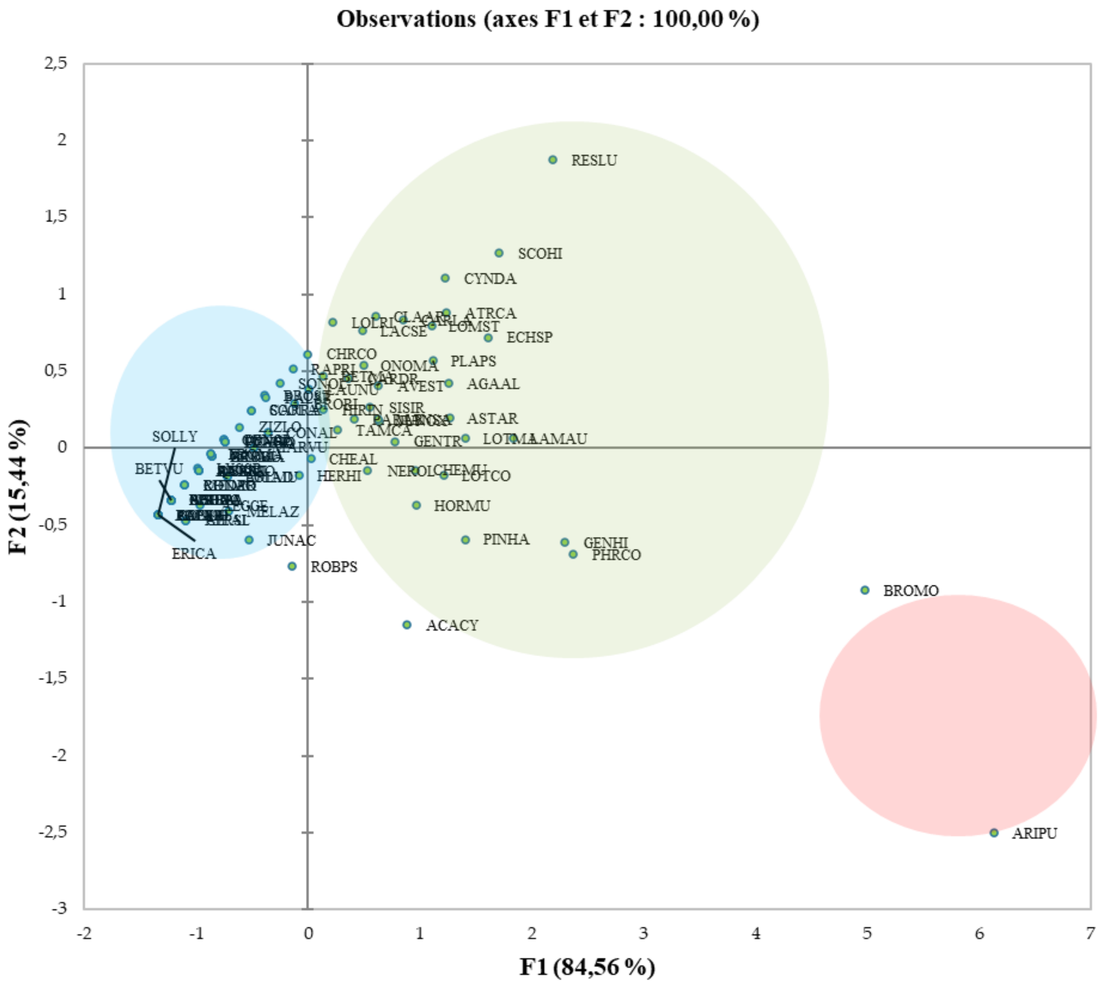

Figure S1. Principal component analysis(PCA) of the vegetation dataset highlighting the relative frequency and the cover values of plant species. Group (**a**) Colored in blue species with the lowest values., Group (**b**) Colored in green species with average values, Group (**c**) Colored in red species of with the highest values

Table S2. The species groups according Ward method cluster analysis for species (abbreviations of species names are listed in table 3).

Groups	Species	Nb of species
**a**	*ACACY, AGAAL, ASTAR, ATRCA, AVEST, BETMA, BRORI, BROST, CARDR, CARLA, CARRA, CHEAL, CHEMU, CHRCO, CLAAR, CONAL, CYNDA, ECHSP, GENHI, GENTR, HERHI, HIRIN, HORMU, JUNOX, LACSE, LAMAU, LAUNU, LOLRI, LOMST, LOTCO, LOTMA, MANSA, MARVU, NEROL, ONOMA, PALSP, PARAR, PHRCO, PINHA, PLAPS, RAPRI, RESLU, SCOHI, SCOLA, SISIR, SONOL, TAMCA, ZIZLO*	48
**b**	*AEGGE, AILAL, AMAAL, AMBLI, ANAMO, ARIAD, ASPAC, ATRGU, ATRSE, BETVU, BRORU, CENCI, CENMA, CERSI, CISTSA, CONAL, CONAR, ECHHO, EROMA, ERICA, CONLI, GLETR, HEYSP, JUNAC, LOLMU, LYGSP, MALPA, MEDPO, MELAZ, PAPRH, PHODA, PISLE, PLACO, POLMO, QUEIL, RETMO, ROBPS, SANMI, SHIBA, SOLLY, Nd*	41
**c**	*ARIPU, BROMO*	2

Notes: Abbreviations of plant species names are listed in Table S1.

Table S3. Bioconcentration Factor (BCF), Translocation Factor (TF), and Biological Accumulation Coefficient (BAC) of the Studied Plant Species.

**Touissit plants**	**BAC**			BCF			TF		
	Cu	Pb	Zn	Cu	Pb	Zn	Cu	Pb	Zn
Aristida pungens	0,17	0,12	0,06	0,38	0,17	0,09	0,44	0,69	0,64
Astragalus armatus	0,2	0,09	0,13	0,13	0,04	0,19	1,53	2,21	0,68
Atractylis caespitosa	0,05	0,12	0,13	0,04	0,12	0,07	1,17	1,01	1,74
Chenopodium murale	0,07	0,12	0,07	0,06	0,08	0,05	1,19	1,43	1,31
Genista hirsuta	0,03	0,02	0,03	0,06	0,04	0,03	0,48	0,47	1,01
Genista tricuspidata	0,07	0,02	0,04	0,17	0,13	0,13	0,41	0,15	0,3
Lotus corniculatus	0,3	0,36	0,25	0,04	0,04	0,03	6,82	8,63	7,27
Lotus maroccanus	0,24	0,23	0,31	0,15	0,13	0,16	1,58	1,72	1,93
Melia azedarach	0,07	0,03	0,02	nd	nd	nd	nd	nd	nd
Pinus halepensis	0,01	0,01	0,01	0,03	0,07	0,05	0,25	0,11	0,28
Pistacia lentiscus	0,11	0,03	0,01	nd	nd	nd	nd	nd	nd
Quercus ilex	0,07	0,04	0,01	nd	nd	nd	nd	nd	nd
Reseda lutea	0,15	0,29	0,22	0,03	0,03	0,04	5,41	8,38	5,8
Retama monosperma	0,01	0,01	**0,01**	**nd**	**nd**	nd	nd	nd	nd
Robinia pseudoacacia	0,02	0	0,01	nd	nd	nd	nd	nd	nd

Notes: Values are calculated with the mean metal's concentrations in plants rhizospherique soil.

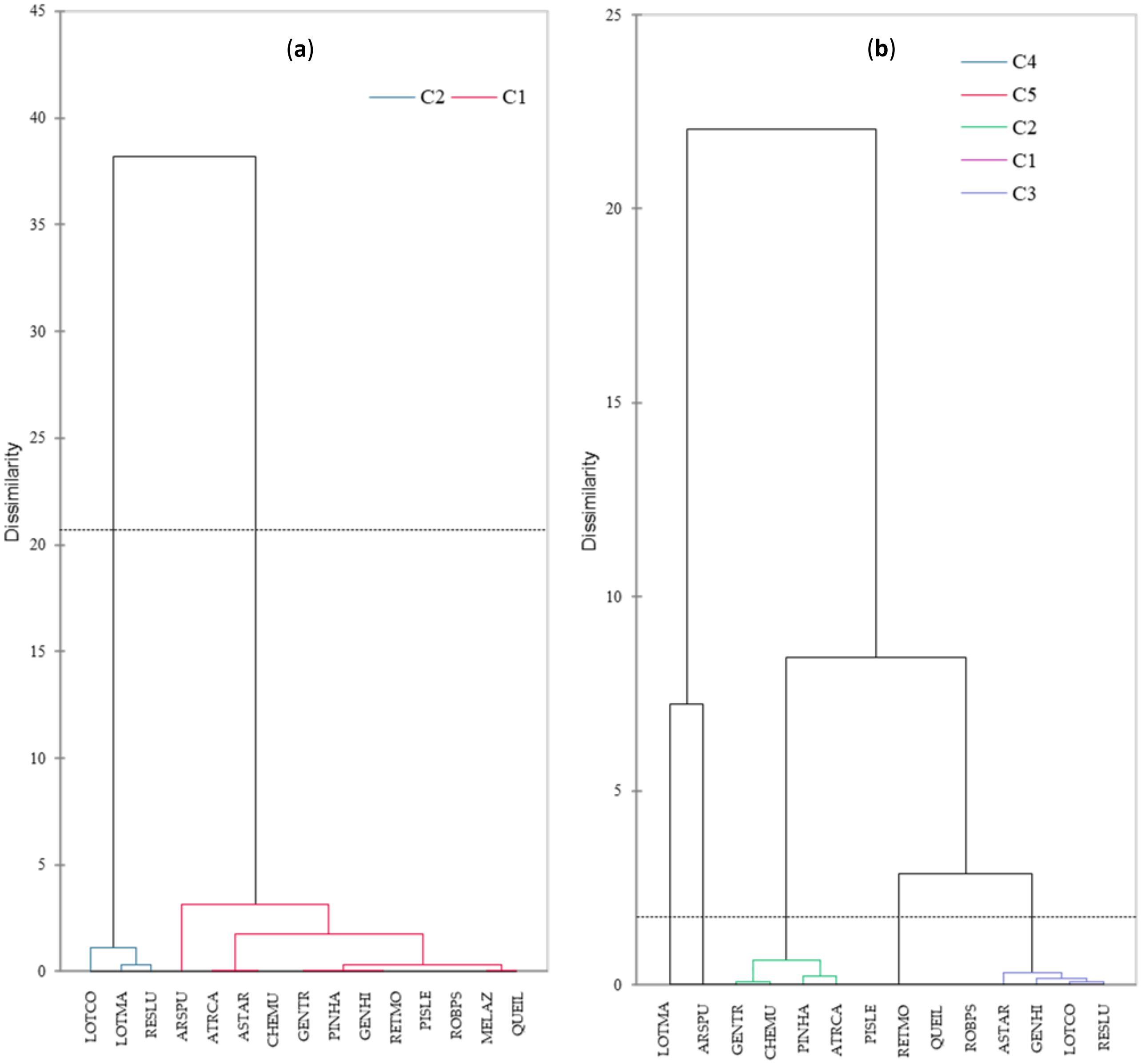

Figure S2. Dendrogram Derived from Ascending Hierarchical Classification (AHC) of Metal Concentrations in Shoots (a) and Roots (b) of Investigated Plant Species: Species Name Abbreviations are listed in Table 2. ***C1: group 1, C2: group 2, C3: group 3, C4: group 4, C5: group5.***

Table S4. Plants phytoremediation strategy

**Species code**	**Phytoremediation Strategy**
ARSPU	Phytostabilisation (Pb, Zn, Cu)
ASTAR	Phytoextraction (Pb), Phytostabilisation (Zn, Cu)
ATRCA	Phytoextraction (Pb, Zn, Cu)
CHEMU	Phytoextraction (Pb, Zn, Cu)
GENHI	Phytostabilisation (Pb,Cu), Phytoextraction (Zn)
GENTR	Phytostabilisation (Pb, Zn, Cu)
LOTCO	Phytoextraction (Pb, Zn, Cu)
LOTMA	Phytoextraction (Pb, Zn, Cu)
MELAZ	Phytostabilisation (Pb, Zn, Cu)
PINHA	Phytostabilisation (Pb, Zn, Cu)
PISLE	Phytostabilisation (Pb, Zn, Cu)
QUEIL	Phytostabilisation (Pb, Zn, Cu)
RESLU	Phytoextraction (Pb, Zn, Cu)
RETMO	Phytostabilisation (Pb, Zn, Cu)
ROBPS	Phytostabilisation (Pb, Zn, Cu)
